# Schlucken und Schluckstörungen im Fokus der HNO-Heilkunde: Von der Diagnostik zur individuellen Therapie

**DOI:** 10.1007/s00106-025-01630-3

**Published:** 2025-04-09

**Authors:** Cornelia Schwemmle, Niall Watt, Christoph Arens

**Affiliations:** Zentrum für Hals‑, Nasen- und Ohrenheilkunde, Universitätsklinikum Giessen, Klinikstraße 33, 35392 Gießen, Deutschland

**Keywords:** Dysphagie, Kopf-Hals-Tumoren, Fluoroskopie, Ösophaguserkrankungen, Pharynxerkrankungen, Dysphagia, Head and neck neoplasms, Fluoroscopy, Esophageal diseases, Pharyngeal diseases

## Abstract

Die Dysphagie bezeichnet die schmerzlose Beeinträchtigung des Nahrungstransports von der Mundhöhle in den Magen, bei Schmerzen beim Schlucken besteht eine Odynophagie. Beschwerden beim Leerschlucken weisen auf einen Globus pharyngis hin. Schluckstörungen können einzelne oder alle Phasen des Bolustransports betreffen. Die Ursachen sind vielfältig, weil der Schluckvorgang die Integrität komplexer Steuerungsareale im Gehirn und der peripheren sensorisch-neuromuskulären Strukturen erfordert. Die Diagnostik besteht aus Anamnese, Screeningbögen, Fragebögen und der flexiblen (video)endoskopischen Schluckdiagnostik als Goldstandard. Flankierend sind u. a. elektrophysiologische Diagnostik und Videofluoroskopien. Die HNO-ärztliche Herausforderung ist bei der operativen Tumorbehandlung im Bereich der oberen Schluckstraße der möglichst große Erhalt der schluckrelevanten Strukturen und posttherapeutisch die Option zu individuellen chirurgischen Maßnahmen für die Schluckverbesserung. Schlucktherapien komplettieren den Behandlungsrahmen.

## Abstract (en)

Dysphagia refers to painless impairment of the transport of liquid or solid food from the oral cavity to the stomach. If pain accompanies swallowing, this is termed odynophagia, and discomfort when swallowing saliva suggests a globus pharyngis. Swallowing disorders can occur during any one or all phases of bolus transport. The causes are manifold, due to the involvement of central control areas within the brain as well as the peripheral nervous system. The diagnosis consists of a detailed case history as well as screening forms and questionnaires. In Germany, the gold standard diagnostic procedure is flexible (video-)endoscopic evaluation of swallowing. Videofluoroscopy and electrophysiological examinations complement the diagnosis. Therapeutic options comprise functional strategies and rehabilitative exercises. However, the most important task of otorhinolaryngologists, in addition to the primary surgical treatment of tumors in the area of the upper swallowing tract (taking into account the preservation of swallowing structures), is the assessment of posttreatment dysphagia with the option of individualized surgical measures to improve swallowing.

## Bedeutung des Schluckens

„Wenn ich gut gegessen habe, ist meine Seele stark und unerschütterlich: Daran kann auch der schwerste Schicksalsschlag nichts ändern.“ (Zitat Jean-Baptiste Molière, 1622–1673). Essen/Trinken sind untrennbar miteinander verbunden und haben seit jeher Dichter, Schriftsteller und Denker beflügelt. Das Schlucken als (über)lebenswichtige Fähigkeit findet im Alltag jedoch erst Beachtung, wenn es nicht mehr richtig funktioniert.

Der Schluckvorgang dient dem aktiven Transport von flüssigen bis festen Konsistenzen aus der Mundhöhle in den Magen sowie dem Schleim der schleimproduzierenden Zellen im Kopfbereich und dem Rachen; er bewerkstelligt die periodische Öffnung der Tuba auditiva [4]. Immerhin schlucken Erwachsene innerhalb von 24 h zwischen 580- bis 3400-mal (im Tiefschlaf keine Schluckbewegungen). Eine hochpräzise zentrale und periphere Koordinationsleistung, anspruchsvolle Anatomie (Überkreuzung Atem- und Schluckwege) und das Zusammenspiel von Willkür- und Reflexmotorik machen das Schlucken zu einem störanfälligen Konstrukt [136]. Ist der geregelte Nahrungstransport gestört, besteht eine Dysphagie.

## Dysphagieursachen

Ihre Ursachen sind die Reduzierung der Effektivität propulsiver Kräfte (Paresen, Operationen) *hinter* dem Bolus und/oder die Widerstandserhöhung *vor *dem Bolus (z. B. Ösophagusstenose). Additiv können beim Schlucken Schmerzen bestehen (Odynophagie) oder Missempfindungen im Larynxbereich (Globus-/oder Kloßgefühl, typischerweise unabhängig von der Nahrungsaufnahme). Die Ursachen einer Dysphagie sind deshalb so vielfältig, weil der Schluckvorgang ein hochkomplexes System aus nervaler, sensorischer, muskulärer und zentraler Koordination ist. Strukturveränderungen der Schluckstraße, insbesondere Tumoren im Halsbereich, sowie alle chirurgischen und konservativen antionkologischen Therapien können das Schlucken kurzfristig und langfristig stören [98].

Auch die Alterung zentraler und peripherer sensorischer, neurologischer und muskulärer Koordinationsprozesse verändert den Schluckvorgang (Presbyphagie). Zeigen sich Veränderungen über die körpereigenen Kompensationsfähigkeiten hinaus, besteht eine Presbydysphagie. Ältere HNO-Tumorpatienten, die bislang ihre Dysphagie kalorisch kompensieren konnten, werden durch ebendiese Alterungsprozesse dann mit einer alltagsrelevanten Gewichtsabnahme konfrontiert: Dies gilt auch für Betroffene, deren Dysphagie andere Ursachen hat. Bei der heute häufig verwendeten leitliniengerechten Medikation einzelner Krankheiten ergibt sich rasch eine Polymedikation mit unüberschaubaren pharmazeutischen Interaktionen, die auch den Schluckvorgang nachhaltig stören.

Viele neuromuskuläre Krankheiten gehen mit einer Dysphagie einher, posttraumatisch nach Schädel-Hirn-Trauma und zerebralen Blutungen sowie Ischämien. Allgemein gehört die Dysphagie zu den bedrohlichsten Symptomen bei neurogenen Störungen [73].

Schluckstörungen begleiten Betroffene nach längerfristigem Intensivaufenthalt und/oder einer längerfristigen Beatmungserfordernis.

Je mehr Faktoren vorliegen, die eine Dysphagie auslösen, umso schwerer präsentiert sie sich. Die Prognose ist vom Alter, dem Schweregrad der Dysphagie, der möglichen Beseitigung von Ursachen und von individuellen Stabilisierungs‑/Regenerierungsressourcen der Betroffenen abhängig. Dysphagiefolgen prägen Malnutrition, Gewichtsverlust, erhöhte Mortalität.

Das Wissen um die Ursache und um mögliche Therapien kurz- und langfristig zur Verbesserung physischer und psychischer Patientenstabilität stellt das ärztliche und therapeutische Personal vor Herausforderungen.

## Schluckentwicklung

Die Essens- und Schluckentwicklung ist ein jahrelanger komplexer Lernprozess, dessen Meilensteine in zeitlich terminierten, sensiblen Lebensabschnitten nacheinander erreicht werden [113].

### Pränatal

In der Embryonalperiode bildet sich in Schwangerschaftswoche (SSW) 3 ein primitiver Mund, in SSW 4 Kiemenbögen und Schlundtaschen, aus denen sich später Gesicht, Nase, Mund, Larynx und Pharynx entwickeln [4]. Ab der 13. SSW sind Schluckbewegungen zu beobachten [113], nach der 20. SSW werden Zungenbewegungen komplexer und ab der 28. SSW anterior-posteriore Zungenbewegungen erkennbar. Aktives Saugen entwickelt sich in der 34.–37. SSW (orale Nahrungsaufnahme meist ab diesem Entwicklungsalter bei Frühgeborenen möglich [113]).

### Postnatal

Postnatal muss erstmals das Schlucken und Atmen koordiniert werden [113], die Anatomie erleichtert den Schluckvorgang: Der harte, zahnlose Gaumen ist relativ flach, Ringknorpel und Kehlkopf stehen weit kranial, sodass sich die Epiglottis hinter den weichen Gaumen schiebt, der Kehlkopf bewegt sich direkt in den Nasopharynx [4]. Außerdem schiebt sich auch in dieser Altersgruppe die Epiglottis über die Glottis, sodass das Atmen in der pharyngealen Phase nicht möglich ist. Zusätzlich besteht ein zentral determinierter Atemstopp beim Schlucken [61, 113]. Die weit verbreitete Meinung, dass ein Säugling gleichzeitig schlucken und atmen kann, ist unrichtig. Frühkindliche Reflexe (z. B. transversaler Zungenreflex/-protrusion) reduzieren sich schrittweise im Verlauf des ersten Lebensjahres durch zentrale/periphere Entwicklungsfertigkeiten und werden danach durch Gehirnreifung, sensorische Reifung, Lernen und Erfahrung zunehmend unterdrückt. Die Akzeptanz und Kompetenz für festere und inhomogene Konsistenzen werden durch das Kauen mit lateral-rotatorischem Kauschlag und die abnehmende Hypersensibilität der mittleren und hinteren Zungenoberfläche gebahnt. Die Dentifikation beginnt im/um den 6. Lebensmonat (Frontzähne des Unterkiefers) und ist vor dem vollendeten dritten Lebensjahr mit 20 Zähnen abgeschlossen [113], es beginnt die Umstellung/Reifung des infantil-viszeralen Schluckmusters auf das somatische Schluckmuster. Mit 4 Jahren ist die Ess- und Schluckentwicklung weitgehend abgeschlossen [113].

## Schluckphasen

Der Gesamtablauf erfordert die bilaterale, koordinierte Aktivierung und Inhibition von mehr als 25 Muskelpaaren in der Mundhöhle, Rachen, Kehlkopf und Speiseröhre. Hierbei sind 5 Hirnnerven (V, VII, IX, X und XII) sowie die Ansa cervicalis (C1–C4) beteiligt [68]. Die überwiegend willkürliche orale Vorbereitungsphase, die orale Phase sowie die reflektorische pharyngeale Phase unterliegen einer somatischen Innervation (quergestreifte Muskulatur). Die Ösophagusperistaltik wird neben intrinsischen Vorgängen durch den „dorsalen Vaguskomplex“ der Medulla oblongata („central pattern generators for swallowing“, CGP) gesteuert, die Innervation der glatten Ösophagusmuskulatur erfolgt durch das autonome Nervensystem [43]. Die zentralnervöse Steuerung umfasst neben den Schluckzentren des Hirnstamms ein ausgedehntes supramedulläres Schlucknetzwerk (einbezogen sind u. a. Insel, Gyrus cinguli, Stammganglien, prämotorische Areale und der primäre sensomotorische Kortex) [68]. Unabhängig von der Händigkeit besteht eine interindividuell unterschiedliche Schluckdominanz im Großhirn, allgemein scheint die linke Hemisphäre eher für die orale Vorbereitungsphase und die orale Phase zuständig [43], die rechte Hemisphäre eher für die pharyngeale Phase [131]. Der Schluckablauf kann in 4, wenn man die präoral-antizipatorische Phase (Überprüfung der Nahrung auf Geruch, Temperatur, Aussehen, sensorische Lippenkontrolle) mitzählt, in 5 Phasen eingeteilt werden [4].

1. Orale Vorbereitungsphase (modifiziert nach [4]):
  Überprüfung/Kontrolle der Nahrung im Mund auf Geruch, Geschmack, Temperatur, Volumen: bei „Missfallen“ Ausspucken des Bolus
  Bolusformung/-platzierung, Zerkleinerung bei inhomogenen, eher trockenen Boli mit Speichelvermischung
  Lippenschluss (M. orbicularis oris, Wangentonus: M. buccinator), seitlich in Mundvorhof ausgewichene Boli: zurückschieben unter Kauleiste, N. facialis (VII): Innervation der mimischen Muskulatur
  Lateral-rotatorischer regelmäßiger Kauschlag (Kinder: 20 Zähne, Wechselgebiss: 32 Zähne mit den 4 Weisheitszähnen) in leicht versetzter Verschlüsselung
  Kaumuskulatur: von Ästen des N. mandibularis innerviert, M. temporalis, M. masseter, M. pterygoideus medialis, auch Vorschubbewegung/Kieferschluss, Kieferöffnung durch M. pterygoideus lateralis, M. geniohyoideus, M. mylohyoideus am knöchernen Unterkiefer
  Zunge formt Bolus, schiebt ihn unter die Zähne (Zungenspitze/Zungenränder schieben sich an Alveolenbasis des Oberkiefers, Zunge macht leicht schiebende Bewegungen), Speichel „weicht“ Bolus zu homogeneren Masse ein, Verhinderung vorzeitigen Abgleitens des Bolus in Pharynx durch Anhebung posterodorsaler Anteile der Zunge und Senkung des Gaumensegels
  Variable Dauer, je nach „anspruchsvoller“ Konsistenz
2. Orale Phase (modifiziert nach [4]):
  Bolustransport vom Mund in Oropharynx, willkürliche Steuerung, Beginn mit Zungenspitzenbewegung, endet mit Auslösung des Schluckreflexes
  Zungenspitze und vordere Zungenränder bilden Zungenfurche für Bolus
  Zungenmuskulatur inseriert im Mundboden (N. hypoglossus), äußere Muskulatur (M. genioglossus, M. hyoglossus, M. styloglossus) und innere Muskulatur (M. longitudinalis, M. verticalis, M. transversus), äußere Zungenmuskulatur zieht bei Kontraktion gesamten Zungenkörper mit Bolus von vorn nach hinten, intrinsische Zungenmuskulatur befördert Bolus mit wellenförmiger Bewegung entlang des Gaumendachs nach hinten
  Beim Kauen Transport des Bolus über V‑förmig abgesenkte Zungenmitte (Zungenfurche) in Valleculae, festere Boli „stauen“ sich im Zungengrund
  Triggerzone Plicae pharyngoepiglotticae, Schluckreflexauslösung
  Kleine Flüssigkeitsmengen zwischen Zungengrund und Gaumensegel können abgleiten in Valleculae ohne Schluckreflexauslösung
  Exspiration: Bolusduftmoleküle erreichen Regio olfactoria, nachfolgend sensorische Informationen über Rezeptoren zum Schluckzentrum, zentrale Regulation
3. Pharyngeale Phase (modifiziert nach [4]):
  Pharynx: etwa 12–15 cm langer Muskelschlauch (Schädelbasis – Speiseröhreneingang), Kreuzung Luft‑/Speiseweg
  Aufhängung elastisch zwischen Schädelbasis und Zwerchfell, Atemweg mit Hyoid und Larynx zwischen Schädelbasis und Mandibula, oberer Thoraxapertur mit Muskeln/Bändern elastisch verbunden
  Pharynx 3 Etagen:
    1. Nasopharynx: von Rachendach bis Gaumensegel (Öffnung über Choanen, Ansatz Tuba auditiva)
    2. Oropharynx: von Gaumensegel nach kaudal bis Plica pharyngoepiglottica
    3. Hypopharynx: Epiglottis, seitlich aryepiglottische Falten zur Incisura interarytaenoidea, paariger Recessus piriformis, seitlich aryepiglottische Falten, kaudal bis oberer Ösophagussphinkter (oÖS), mittig Larynxtrichter
  Pharynxschlauch: 3 „Schlundschnürer“ (Mm. constrictores pharyngis, Schlundheber Mm. levatores pharyngis), M. constrictor pharyngis superior (N. glossopharyngeus, N. IX) wölbt sich beim Schlucken als sog. Passavant-Ringwulst Gaumensegel entgegen, nachfolgend Nasopharynxabschluss
  Zirkulärer Muskelzug vor den Nasengängen „Rhinosphinkter“ (< 10 % bei HNO-Patienten [15]), schließt kurz vor Velumverschluss, öffnet kurzzeitig nach Relaxation, weitere Muskeln (M. constrictor pharyngis medius, Plexus pharyngeus, Nn. glossopharyngeus und vagus, N. IX und N. X, M. constrictor pharyngis inferior, N. X) unterstützen Vorschieben des Bolus
  Bolustransport durch Rückwärtsbewegung der Zunge in Hypopharynx, Hyoidbewegung nach kranial/ventral
  Larynxelevation/ventral durch M. thyrohyoideus, Öffnung des Hypopharynx mit Sogwirkung, gleichzeitig Verschluss mit Gaumensegelschiebung an Rachenhinterwand, Epiglottis schließt über Larynxeingang, Verengung supraglottischer Raum, Stimmlippen/Taschenfalten schließen sich, Aryknorpel kippen ins Lumen, Epiglottis hebt sich mit Kontraktion M. thyrohyoideus, senkt gleichzeitig Oberrand gegen Pharynxhinterwand, um Bolus aufzufangen
  Schluckreflexauslösung durch Boluskontakt an vorderen Gaumenbögen, Rachenhinterwand, Zungengrundmukosa, Recessus piriformis oder Postkrikoidalmukosa [15]
  Schluckreflexzeitpunkt inter-/intraindividuell, unwillkürlich, Triggerung taktil
    Wässrige Boli kategorisch in Sekundenbruchteilen abgeschluckt
    Dickflüssige/feste Speiseanteile: Ansammeln in Valleculae, stufenweise oder kaskadenförmiges Abschlucken, beim Schlucken reflektorischer Atemstopp
  Abschluss pharyngeale Phase: Öffnung oÖS, Verschlusszone oÖS durch M. cricopharyngeus und kaudale Teile Pars thyropharyngea unterer Schlundschnürer, kraniale Abschnitte zervikale Ringmuskelschicht Ösophagus und durch submuköse Venengeflechte [56].
  oÖS: Relaxation, dann Öffnung, dann Erweiterung der Öffnung, danach Kollaps und erneuter Verschluss. oÖS relaxiert etwa 0,1 s vor Kehlkopfhebung, durch Kontraktion M. thyrohyoideus mit leichtem Kippen und Aufsteigen Epiglottis (Elevation), gefolgt von Vorwärtsbewegung Larynx nach vorne oben, Konstriktormuskulatur drückt mit Zungenstempel freien Epiglottisrand nach unten, Bolus erreicht obere Speiseröhre, Bolusdruck beeinflusst Weite der oÖS-Öffnung
  Sobald Bolus im Ösophagus ist und Larynx/Os hyoideum Ruheposition einnehmen, schließt sich oÖS [56], Rückstellung Epiglottis, erneute Glottisöffnung, Abschluss pharyngeale Phase
4. Ösophageale Phase (modifiziert nach [4]):
  Ösophagus (Beginn Höhe 6. Halswirbel, etwa 25 cm langer Muskelschlauch mit Mündung spitzwinklig im Magen), Innervation N. vagus über Plexus oesophageus und Truncus sympathicus, Peristaltik durch N. vagus verstärkt, durch Sympathikus gehemmt
  Ösophagus: 3 anatomische Engstellen: 1. Ringknorpel, gleichzeitig engste Stelle insgesamt (etwa 15 mm Durchmesser), 2. Aortenbogen, 3. Zwerchfelldurchtritt
  oberstes Drittel quergestreifte/im mittleren Bereich zusätzlich glatte, untere Hälfte nur glatte Muskulatur; Muskelfasern verlaufen außen schraubenförmig (äußere Längsmuskelschicht) und innen schräg oder zirkulär (innere Ringmuskelschicht) [50], Peristaltik beinhaltet primäre und sekundäre Welle: mechanische Reizung (Speisereste Ösophaguswand) löst sekundäre Welle aus (Reinigungswelle). In Tab. 1 Zusammenfassung der Schluckphasen.

**Tab. 1:** Schluckphasen. (Nach [108])

Phase	Funktionsablauf	Dauer	Motorische Steuerung
1. Orale Vorbereitungsphase	Aufnahme, Boluszerkleinerung, Durchmischung mit Speichel, Bolusformung durch Zunge, Sammlung in der „Zungenschüssel“, Abschließen Mundhöhle durch Velum-Zungenbasis-Kontakt	Variabel (abhängig von Beschaffenheit Nahrung usw.)	Willkürlich
2. Orale Transportphase	Bolustransport in hintere Mundhöhle durch Anhebung, Rückwärtsbewegung Zunge	≤1 s	Willkürlich
3. Pharyngeale Phase	Auslösung Schluckreflex Bolustransport Pharynx durch Stempeldruck Zunge und Pharynxperistaltik, Verschluss Nasopharynx, Anhebung/Vorwärtsbewegung und Verschluss Larynx, Öffnung oberer Ösophagussphinkter (oÖS)	≤1 s	Reflektorisch
4. Ösophageale Phase	Bolustransport durch peristaltische Wellen, Öffnung unterer Ösophagussphinkter (uÖS)	4–20 s	Reflektorisch

## Schluckstörungen

### Lokalisation

Pharynx und Ösophagus bilden eine funktionelle Einheit. In der Diagnostik werden Pharynx und Ösophagus aber meist getrennt voneinander betrachtet, was differenzialdiagnostisch hilfreich ist. Allgemein wird zwischen (oro)pharyngealer und (gastro)ösophagealer Dysphagie unterschieden ([136]; die Tab. 2 zeigt die Zuordnung von Symptomen zur Lokalisation der Schluckstörung).

**Tab. 2:** Symptome oropharyngealer und ösophagealer Dysphagie (Mod. nach [85])

Oropharyngeal	Drooling: Bolusbestandtreile kommen/laufen aus dem Mund heraus
	Mangelnde orale Boluskontrolle und Transport
	Schwierigkeiten, Schluckvorgang einzuleiten
	Nasale Penetration
	Steckenbleiben von Nahrung im Hals, Husten, Räuspern, Regurgitation
	Änderung Stimmklang während oder direkt nach Essen, Trinken oder Medikamenteneinnahme
	Verlängerte Essensdauer
	Verstärkte Verschleimung, gehäufte Infekte, Pneumonien
	Gewichtsabnahme
Ösophageal	Halsschmerzen, Globusgefühl, thorakale Schmerzen, Brennen Herzregion Regurgitation von Speisen und Tabletten Rezidivierende Pneumonien Hustenattacken beim Hinlegen nach dem Essen „Feststecken“ von Nahrung im Hals, hinter dem Brustbein Gewichtsabnahme

Allgemein können sämtliche Veränderungen der Oberfläche oder tieferliegender Strukturen Schluckstörungen verursachen oder verstärken:

- Mukositiden, Ulzera, narbige Veränderungen und Xerostomie mit/oder ohne zähen Mukus [96], Rigidität Larynxskelett (z. B. Epiglottisverplumpung)
- Paresen, zentral/peripher: Stimmlippenparesen mit Glottisinsuffizienz, bei Lähmungen von N. glossopharyngeus, N. vagus, N. hypoglossus [66].
- Hyperkinesen: Kleinhirn- und/oder Hirnstammläsionen verursachen Koordinationsstörungen im Schluckvorgang [68].
- Mukosale Sensibilitätsstörungen: Beeinträchtigung pharyngeale Triggerung, häufig Penetration mit gurgelnder Stimme sowie Speichel‑/Sekretansammlung in Hypopharynxstrukturen [4, 83].
- Ösophagusspasmen, Myotonien, Vaskulitiden, Kollagenosen stören Motilität, Koordination der Ösophagussphinkter und Propulsionswelle.
- Krikopharyngeale Funktionsstörungen: Koordinationsstörung pharyngeale Propulsion, fehlende ösophageale Relaxation (z. B. Hirnstammläsionen, Parkinson-Syndrom, Myopathien), Bolusretention im krikopharyngealen Übergang [127].

Die *retrograde* krikopharyngeale Dysfunktion, bereits 1987 beschrieben, steht erst in neuerer Zeit im Fokus der internationalen Literatur. Leitsymptom ist die Unmöglichkeit des Aufstoßens von geschluckter Luft mit Luftvöllegefühl („kann nicht rülpsen“) durch mangelhafte postdeglutitive Relaxation des oÖS [80].

### Ursachen

#### Anatomisch-funktionelle Störungen

Angeborene Fehlbildungen (z. B. Lippen-Kiefer-Gaumen-Spalten), angeborene Lähmungen der Gesichtsmuskulatur und andere Dysformationen, z. B. bei genetischen Syndromen, stören Schluckeffizienz und Bolustransport.

Sämtliche neurologische Krankheiten können eine Dysphagie verursachen. Bezüglich Störungen in der Gehirnfunktion zeigen sich bei mindestens 50 % der Patienten mit ischämischem oder hämorrhagischem Apoplex Schluckstörungen [35], bei Patienten mit Schädel-Hirn-Trauma in 60 % der Fälle [81], Patienten mit neurodegenerativen Gehirnveränderungen (z. B. Demenz, M. Parkinson) sind häufig betroffen. Daneben ist bei neuromuskulären Krankheiten die Dysphagie häufiges Symptom, bei Betroffenen mit amyotroper Lateralsklerose annähernd bis zu 100 %. Muskelkrankheiten wie genetische Myopathien, Polymyositiden und die Einschlusskörperchenmyositis sind häufig mit Schluckbeschwerden verbunden [34].

Sämtliche Tumoren in der Schluckstraße werden früher oder später das Schlucken beeinträchtigen, das Dysphagieausmaß wird durch Größe, Tumorausdehnung und Infiltration in das umgebende Gewebe maßgeblich beeinflusst. Speziell Hypopharynxtumoren bleiben lange Zeit unentdeckt und können sich, da sie keine anatomischen Barrieren haben, ungehindert ausbreiten.

Postoperativ provozieren häufig Substanzverlust/veränderte Anatomie und Nervenschäden (z. B. N. recurrens) Schluckstörungen. Resektionen schluckrelevanter Strukturen (Epiglottis, aryepiglottischen Falte) sowie Narbenbildung und chronisches Ödem in der Schluckstraße führen meistens zu lebenslangen Schluckstörungen. Auch nach Laryngektomie können Bolustransportstörungen durch narbige Strikturen in der Schluckstraße auftreten, Leckagen am Stimmventilkanal provozieren Aspirationen in die Trachea [129].

Die Radiatio im Mund/Pharynx‑/Larynxbereich verursacht Schluckstörungen vorrangig in den ersten Monaten nach Therapieabschluss durch Ödeme. In der chronischen Phase überwiegen Neuropathien und Fibrosen mit Rigidität v. a. der Larynxstrukturen [102]. Kombinationen von Radiatio und Chemotherapie verursachen häufiger Dysphagien als die alleinige Radiatio [87], daneben sind Alter, fortgeschrittenes Tumorwachstum, Hypopharynxlokalisation, prätherapeutische Dysphagie sowie Alkoholabusus Faktoren für den Schweregrad der Dysphagie [41].

In den vergangenen Jahren zeigte sich eine große Dynamik bei Immuntherapien mit Checkpointinhibitoren als eine neue Behandlungsoption bei Kopf-Hals-Tumoren [31, 139]. Checkpointinhibitoren sind Antikörper, die den Tumorsignalen zur Reduktion der Immunantwort entgegenwirken. Von besonderer Bedeutung ist aktuell der PD1-Rezeptor („programmed cell death“), der die T‑Zell-Aktivität herunterregelt. Durch eine Blockade der Interaktion zwischen PD1-Rezeptor und Ligand („PDL1“) können PD1-Inhibitoren die T‑Zell-Reaktion gegen den Tumor verstärken [22]. Checkpointinhibitoren unterscheiden sich hinsichtlich ihres Nebenwirkungsprofils von Chemotherapeutika: Die Toxizität von ist vergleichsweise niedrig, kann aber alle Organsysteme betreffen und auch noch sehr spät nach Therapiebeginn auftreten [31]. Am häufigsten betroffen sind Haut (Exanthem), Darm (Kolitis), endokrine Organe (Thyreoiditis, Hypophysitis), Leber (Hepatitis) und Lunge (Pneumonitis) [139]. Inwiefern langfristig Mukositiden der Schluckstraße auftreten können, ist bisher ungeklärt, die aktuellen Erfahrungen weisen jedoch nicht darauf hin.

#### HWS-bedingte Schluckstörungen

Schluckstörungen mit/ohne Globusgefühl werden häufig durch eine funktionell-reflektorische Störung der Halswirbelsäule (HWS) verursacht, z. B. durch Schleudertraumata. Höhenlokalisation sind meistens Blockaden von C2/3 und C3/4 [80].

Degenerative HWS-Veränderungen/Exostosenbildung können durch „balkonartige“ Vorwölbungen der Pharynxhinterwand den Bolustransport stören (Einschränkung der Bewegung von Epiglottis, Aryknorpel, Larynxelevation) [105, 125], bleiben aber in etwa 30 % völlig asymptomatisch und werden als Zufallsbefund z. B. bei bildgebender HWS-Diagnostik zufällig entdeckt [136]. HWS-Spondylodesen führen durch das Operationstrauma und die Verminderung der natürlichen Lordose häufig zu langfristigen/chronischen pharyngealen Transportstörungen (zervikal-anteriorer Zugang häufiger als posterior) [40].

#### Kindliche Schluck‑/Fütterstörungen

Dysphagien können im Säuglings‑/Kleinstkindesalter häufig nicht von Störungen des Essverhaltens unterschieden werden [113], sodass der Begriff Ess‑/Schluckstörung in der Literatur synonym verwendet wird. Kindliche Dysphagien sind bedingt durch traumatische Hirnschäden und -blutungen, neuromuskuläre Ursachen (z. B. infantile Zerebralparese, spinale Muskelatrophien [82]), Frühgeburtlichkeit (z. B. sensomotorische Defizite), Refluxkrankheiten sowie (immunvermittelte) Mukositiden (z. B. eosinophile Ösophagitis) [42] und anatomische Veränderungen wie kraniofaziale Fehlbildungen (Lippen-Kiefer-Gaumen-Spalten, Ösophagusstenosen, auch nach Ösophagusatresien). Auch eine durch orale Medikamentengabe verursachte Schädigung des Ösophagus („oral medication-induced esophageal injury“, OMIEI) kann bei Kindern vorliegen, wenn langfristig oral Medikamente verabreicht werden (z. B. bei Autoimmunkrankheiten). Häufig kann eine OMIEI durch unspezifische Verhaltensveränderungen mit wählerischem/ungenügendem Essverhalten als charakterliche Eigenart fehlinterpretiert werden. Gedeihstörungen und rezidivierendes Erbrechen sind u. U. die einzigen Anzeichen [42]. Frühgeborene (auch ohne Komplikationen) haben ein erhöhtes Risiko für Dysphagien über das Kleinstkindalter hinaus [113]. Allgemein zeigen deutlich entwicklungsretardierte Kinder nicht selten lebenslang Kau- und Schluckschwierigkeiten, die eine kalorisch ausreichende orale Ernährung nicht sicher gewährleisten. Die Dysphagie bleibt durch die Persistenz primitiver Reflexe existent, weil die Entwicklung physiologischer Schluckmuster gestört wird. Bei Kindern mit Syndromen (z. B. Trisomie 21) sind Schluckstörungen in mehr als 50 % vorhanden [23]. Die Auswirkungen sind eine eingeschränkte Lebensqualität, Abhängigkeit von Sondenernährung und Trachealkanülen, Aspiration, pulmonale Erschöpfung [130]. Dehydratation/Malnutrition können für das reifende Gehirn negative Folgen haben und sind vital bedrohlich [82].

Zeichen einer Dysphagie sind Erbrechen oder Regurgitation, Husten während/nach Nahrungsaufnahme und längere Passagezeiten während der oralen Phase, sekundär durch mangelndes Interesse am Essen, körperliche Anspannung, Ausspucken von Nahrung, gurgelnde Atemgeräusche oder eine belegte Stimme. Die Anzeichen können subtil sein oder bei stiller Aspiration auch fehlen [100]. In Tab. 3) gibt es eine Übersicht über die Ursachen kindlicher Schluckstörungen.

**Tab. 3:** Verschiedene Ursachen kindlicher Schluckstörungen (mod. nach [12, 113])

Anatomische Ursachen	Genetische Ursachen	Neurologische Ursachen	Andere Ursachen
– Velumspalten/Lippen-Kiefer-Gaumen-Spalten – Zungenfehlbildungen – Mikro‑/Retrognathie – Choanalatresie – Laryngomalazie – Tracheoösophageale Fistel – Stenosen/Atresie des Ösophagus/Larynx	– Trisomie 21 – Trisomie 18 – Möbius-Sequenz – Apert-Syndrom – Prader-Willi-Syndrom – Pierre-Robin-Sequenz – Beckwith-Wiedemann-Syndrom – Cri-du-chat-Syndrom – CHARGE-Syndrom – Treacher-Collins-Syndrom – 22q11-Mikrodeletion – Cornelia-de-Lange-Syndrom – Meningomyelozele mit Arnold-Chiari-II-Malformation	– Hirn‑/Rückenmarkfehlbildungen – Intrauterine Intoxikationen *Perinatal:* – Hypoxisch-ischämische Hirnschädigung – Frühgeburtlichkeit *Postnatal:* – Tumoren – Infektionen (z. B. Meningitis, Enzephalitis, Polymyelitis) – Schädel-Hirn-Trauma – Bilirubinämie – Degenerative Hirnerkrankungen – Metabolische Enzephalopathien – Erkrankung zentrales Nervensystems (z. B. Zerebralparesen) – Myasthenia gravis	– Medikamente – Eosinophile Ösophagitis – Immunschwäche – Langfristige Sondenernährung – Obstruktion obere Atemwege – Tracheotomie – Langzeitbeatmung/Intubation – Intrauterine Wachstumsretardierung – Intrauterine Infektion – Gastropharyngealer Reflux

*CHARGE* Kolobom, Herzfehler, Atresie der Choane, retardiertes Wachstum, Ohrfehlbildungen

#### Presbydysphagie

Sind die Kompensationsfähigkeiten bei altersphysiologischen Schluckveränderungen (Presbyphagie) erschöpft, besteht eine Presbydysphagie, die typischerweise häufig um das 65. Lebensjahr beginnt [123] (erste merkbare Veränderungen der Schluckkoordination bestehen anekdotisch bereits mit Ende der 4. Lebensdekade). Die Verringerung von Riechen und Schmecken, degenerative Gehirnveränderungen, Sarkopenie und die Reduktion neuromuskulärer Koordinationsprozesse können alle Schluckphasen betreffen [55], speziell die pharyngoösophageale Phase kann um das 3‑ bis 4‑Fache verlängert sein [49]. Die Tab. 4 zeigt altersabhängige Veränderungen innerhalb der Schluckphasen.

**Tab. 4:** Altersabhängige Veränderungen innerhalb der Schluckphasen (Mod. nach [55])

Orale Phase	Pharyngeale Phase	Ösophageale Phase
– Schmeck‑/Riechverminderung – Verminderung oraler Sensibilität – Einschränkung der Kaufunktion – Reduzierte Speichelproduktion – Einschränkung orofazialer Motorik – Abnahme der Zungenkraft – Verzögerte Triggerung der pharyngealen Phase	– Erweiterte Pharynxstrukturen – Verringerte Pharynxstabilität – Larynxelevation verändert – Verspätet einsetzende Hyoidverlagerung – Reduzierter Ruhetonus oÖS – Reduzierte Öffnung oÖS – Reduktion maximaler Krafterzeugung – Verlängerte pharyngeale Transitzeit – Mukosasensibilitätsveränderung	– Erweitertes Ösophaguslumen – Rigide Ösophaguswand – Abnahme Ganglienzellen Plexus myentericus – Reduzierte Ösophagussensibilität – Sekundäre Ösophagusperistaltik reduziert oder ausbleibend – Eingeschränkte Funktion uÖS

*oÖS* oberer Ösophagussphinkter, *uÖS* unterer Ösophagussphinkter

Ältere Patienten haben darüber hinaus vermehrt Begleitkrankheiten, die additiv das Schlucken negativ beeinflussen (z. B. Polyneuropathien) [85].

#### Medikamentenassoziierte Dysphagien

Viele Patienten haben, unabhängig vom Alter, häufig Probleme mit dem Schlucken von Tabletten. Die Schwierigkeiten sind mit Dicke, Länge und Größe der Tabletten assoziiert, Kapseln, die häufig auf der nachgetrunkenen „Wasserwelle“ schwimmen, werden am häufigsten als problematisch genannt [106]. Berichtete Schluckprobleme sind: Hängenbleiben im Hals, Missempfindungen, mehrfaches Nachschlucken erforderlich [106].

Aufgrund fehlender Alternativen werden Tabletten deshalb häufig von Patienten geteilt, gemörsert, in Wasser aufgelöst oder gekaut, mitunter mit deutlichen Folgen einer Mukositis, der Aspiration von Einzelteilen und Tabletteninkarzerierungen (z. B. Sinus piriformis, Vallecula, Ösophagus) [106]. Annähernd alle Medikamente haben durch ihre pharmakologischen Wirkprofile die Potenz, eine Dysphagie auszulösen oder zu verstärken. Dies wird häufig nicht erkannt, anamnestisch nicht eruiert oder stillschweigend akzeptiert [112]. Überdosierungen bei nicht beachteter reduzierter Leber- und Nierenleistung mit Kumulationseffekten können Schluckstörungen initiieren oder verstärken, Arzneistoffe untereinander kumulativ-verstärkende Effekte haben [58]. Ein reduzierter Allgemeinzustand, ein erhöhtes Alter, multiple Medikationen, degenerative Hirnveränderungen und/oder psychiatrische Auffälligkeiten sind Risikofaktoren [98]. Bei medikamentenassoziierten Nebenwirkungen unterscheidet man *direkte* und *indirekte* Wirkungen. Die medikamentöse *direkte *Wirkung verursacht Störungen der Schluckstrukturen (z. B. Mukosaverätzung durch inkarzerierte Tabletten), die *indirekte* Wirkung, wenn der Schluckvorgang in seinem Ablauf gestört wird (z. B. medikamentös verursachte Xerostomie) [124]. Arzneistoffe können durch den *direkten* Kontakt mit der Ösophagusmukosa zu lokalen Entzündungen/Ulzerationen führen und werden als Symptomkomplex unter dem Begriff „oral medication-induced esophageal injury“ (OMIEI) oder „drug-induced esophageal injury“ (DIEI) zusammengefasst. Leitsymptome sind Dysphagie, Fremdkörpergefühl, und/oder eine Odynophagie mit „Hängenbleiben von Nahrung“. Wirkstoff, Art und Größe der Tabletten, Einwirkzeit und die verwendete Flüssigkeitsmenge haben einen Einfluss auf eine OMIEI [112]. Eine Übersicht der Medikamente, die vorrangig eine OMIEI auslösen können, zeigt Tab. 5.

**Tab. 5:** Medikamente, die eine „oral medication-induced esophageal injury“ (OMIEI) auslösen können. (Mod. nach [98, 121])

Antibiotika (Tetrazykline, Doxycyclin, Trimethoprim-Sulfamethoxazol, Clindamycin)
Nichtsteroidale Antirheumatika (NSAR), Acetylsalicylsäure (ASS)
Bisphosphonate
Sildenafil (Phosphodiesterase-5-Hemmer, Vasodilatator)
Kalium
Theophyllin
Chinidin (Antiarrhythmika)
Eisensulfate (in Eisenpräparaten), Vitamin C

Substanzgruppen mit *indirekter* Wirkung sind vorrangig Wirkstoffe, die die zentrale Erregbarkeit und Vigilanz bewusst reduzieren wie z. B. Antikonvulsiva, Antidepressiva. Antiallergika sowie Analgetika (z. B. mit opiatähnlicher Wirkung) können durch ihre sedierende Komponente Schluckfunktionen negativ beeinflussen. Bei Benzodiazepinen wird eine Auswirkung auch auf die laryngeale Schluckaktivität vermutet [91], Nitrazepam (Behandlung von kindlichen Epilepsien) kann durch Muskelkoordinationsstörungen der krikopharyngealen Region zu Aspiration mit Todesfolge bei Kindern führen [69]. In Tab. 6 sind die wichtigsten Medikamente zusammengefasst, die durch eine zentrale Wirkung Dysphagien auslösen/verstärken können.

**Tab. 6:** Zentral wirksame Medikamente, die eine Dysphagie auslösen oder verstärken können. (Nach [85, 98])

Antipsychotika (Haloperidol, Risperidon u. a.)
Anticholinergika
Opioide, Morphine
Antiepileptika
Antidepressiva
Analgetika (vorrangig Opioide)
Antitumormedikamente (Vincristin und andere Alkaloide)
Antihypertensiva
Antiemetika (Metoclopramid)

Fettsenker (Statine) sowie Colchicin [39] sind Auslöser von Myopathien, klinisch kann eine dysphagieverstärkende Wirkung auch schon in geringen Dosierungen bestehen. Kortikosteroide induzieren am häufigsten eine Myopathie [110]. Botulinumtoxine, bei spasmodischer Dysphonie oder zervikaler Dystonie Goldstandard, können durch Diffusion Schluckschwierigkeiten verursachen [111]. Oberflächenanästhetika (in manchen Lutschpastillen enthalten) zur Reduktion von Halsschmerzen, Schmerzen bei Aphtose oder vor geplanten Endoskopien der oberen Luft- oder Speisewege reduzieren die Oberflächensensibilität reversibel. Sie spielen eine Rolle insbesondere bei Tumorpatienten während der Bestrahlung im Halsbereich, wenn sie vor den Mahlzeiten bei Odynophagie Gurgellösungen, z. B. mit Tetracain, verwenden.

### Begleitsymptome

#### Xerostomie

Die Xerostomie ist die vorrangige periphere Nebenwirkung zentral wirkender Medikamente, insbesondere trizyklischer Antidepressiva (z. B. Amitriptylin), Serotonin-Wiederaufnahmehemmer [98] und opiathaltiger Schmerzmittel [54]. Eine medikamentös induzierte Xerostomie hat v. a. bei Älteren beträchtliche Auswirkungen auf den Bolustransport [89]. Die Tab. 7 zeigt eine Zusammenstellung von Medikamenten, die typischerweise eine Xerostomie auslösen können.

**Tab. 7:** Medikamente, die eine Xerostomie auslösen können. (Mod. nach [85, 121])

Anticholinergika: Atropin, Scopolamin (transdermale Pflaster, Tropfen) u. a.
Blutdruckmedikamente (Alpha-Blocker, ACE-Hemmer, Angiotensin-II-Rezeptor-Blocker, Antiarrhythmika)
Disopyramid
Mexiletin
Ipratropiumbromid
Antihistaminika
Diuretika
Opiate
Antidepressiva, Antipsychotika
Retinoide

Die Konstellation einer Polypharmazie mit einer Dysphagie bei marantischer Parotitis, eventuell auch bereits vorliegender Anlage einer perkutanen endoskopischen Gastrostomie (PEG) sind häufige Realität. Hier haben alle die Aufgabe, Medikamente kritisch zu prüfen.

Andere Ursachen einer Xerostomie sind Diabetes mellitus, Bestrahlung (> 25 Gy irreversible Schädigung), Autoimmunkrankheiten (z. B. primäres/sekundäres Sjögren-Syndrom), Sarkoidosen, Amyloidosen u. a. [63].

#### Hypersalivation

Eine Hypersalivation kann Speichelaspirationen unterhalten bei vorbestehender Schluckstörung wie z. B. bei zentral-neurologischen Störungen (M. Parkinson, infantile Zerebralparese u. a.), peripheren Nervenläsionen, Medikamentennebenwirkungen (Neuroleptika u. a.). Ursachen einer Pseudohypersalivation sind vorrangig orofaziale Dysfunktionen, Speichelfisteln, sensorische Störungen im Mund‑/Pharynxbereich. Eine Imbalance durch reduzierte Schluckfrequenz/Schluckeffizienz und mundmotorische Einschränkungen ist bei Kindern mit zerebralen Paresen häufig auch im Erwachsenenalter existent.

#### Aspiration

Letztendlich sind Auswirkungen sowie körperlich/immunologische Strategien bei einer Aspiration bis heute nicht ausreichend geklärt. In allen Lebensdekaden sind Aspirationen nachweisbar [14]. Die „Aspirationspneumonie“ ist multidimensional, die Dysphagie nicht stärkster Prädiktor für eine Aspirationspneumonie [64]. Eine ambulant erworbene Pneumonie und eine Aspirationspneumonie können klinisch nicht voneinander unterschieden werden [119], kein bildgebendes Verfahren kann eine Aspirationspneumonie von einer nichtaspirationsbedingten Pneumonie abgrenzen [51]. Einige Autoren bezeichnen deshalb die Pneumonie beim Älteren eher als Frailty-assoziierte Pneumonie, bei Kaustörungen als Oral-Frailty-Pneumonie [119]. Zumindest sind neben der Aspiration pulmonale Clearance, Bakterienlast und immunologische Prozesse wichtigere Ursachen für eine Pneumonie [92]. Signalkaskaden nach Apoplex initiieren multiple immunologische Vorgänge direkt nach dem Ereignis (Interleukin[IL]-10, Neutrophilenstimulation), Tage bis Wochen danach Aktivierung von IL‑4, IL-10, IL-13, Neutrophilen, Monozyten, NK-Zellen auch im Lungengewebe. Die Überaktivierung des autonomen Nervensystems führt zu Funktionsstörungen von Monozyten, Lymphozyten und zur Einschränkung der mukoziliären Clearance. Ein Schlaganfall induziert Immundepression und ist neben einer Schluckstörung Hauptursache für Letalität und Langzeitmorbidität [37]. Diese identifizierten Faktoren, die eine Pneumonie mitverursachen, geben Hinweise darauf, dass der Begriff der häufig spekulativ bemühten „Aspirationspneumonie“ verlassen werden sollte [37].

## Diagnostik

### Anamnese

Die Anamnese spielt die Schlüsselrolle in der Einschätzung des Ausmaßes und der Art der Ess‑/Schluckstörung. Die Begleitung von Angehörigen, Intensivpflegenden, Therapeuten u. a. sind willkommen, weil sie Einblick in die Lebenssituation des Betroffenen haben und zusätzliche Informationen geben können. Die Anamnese beinhaltet auch die (mutmaßliche) Ursache der Schluckproblematik vonseiten des Patienten, die Dauer, die Persistenz/Progredienz, Regredienz und abschließend die Frage nach Techniken, die der Betroffene vielleicht umsetzt (Kostform, Kopfhaltung usw.).

Die Anamnese für (internistische, psychiatrische) Begleitkrankheiten und die Dokumentation der *vollständigen* Medikamenteneinnahme (inklusive Medikamente, Nahrungsergänzungsmittel und Wirkstoffe wie Kardamomextrakte usw.). Dazu gehört auch die *präzise* Erfassung des Nikotinkonsums (seit wann, wieviel/Tag) und des Alkoholkonsums, das viele Patienten vage nur als „normal“ betiteln. Die Erfassung von Voroperationen runden die Anamnese ab.

Die spezielle Anamnese beinhaltet [4, 113]:

- Wie lange dauern Mahlzeiten (Kinder: mehr als 30 min)?
- Atemprobleme während/nach dem Essen
- Stimme, Sprache, Sprechen
- Körperkontrolle
- Allgemeinzustand
- Bewusstseinszustand (eher durch Fremdanamnese zu beurteilen)
- Bei Kindern: Gewichtszunahme/-stagnation, passen Alter/Größe/Gewicht zusammen? Stresst essen das Kind (und/oder den Erwachsenen)? Schnullerverwendung, Bruxismus (auch ein Zeichen für Schwerhörigkeit), kategorische Nahrungsmittelvorlieben (wählerisches Essverhalten typisch bei Störungen aus dem autistischen Formenkreis)
- Bei Erwachsenen: Gewicht stabil, Abnahme/Zunahme?
- Bestehen/bestanden pulmonale Infekte, wie häufig?
- Ernährung: oral, parenteral, Nasogastralsonde/PEG/perkutane endoskopische Jejunostomie (PEJ) sowie Kombinationen
- Bei Trachealkanülen: seit wann, Grund
- Bisherige Therapie (alle Therapieverfahren)
- Häusliche Situation (Heimbetreuung, Intensivpflege zu Hause, spezielle Einrichtungen)

Die Anamnese über Kostform/Ernährung kann über den Bogenhausener Dysphagiescore präzisiert werden: Er erfasst die Fähigkeit des Speichelschluckens (BODS-1) und der Nahrungsaufnahme (BODS-2). Die Skalen können einzeln oder als Summe zur Schweregradbeurteilung der Dysphagie verwendet werden und eignen sich für Patienten mit/ohne Trachealkanülen (Tab. 8, 9 und 10). Reliabilität und Inhaltsvalidität des BODS wurden nachgewiesen [122].

**Tab. 8:** Bogenhausener Dysphagiescore‑1, BODS‑1 (Speichelschlucken)

Score	Charakteristika
1	Keine Trachealkanüle, effizientes Speichelschlucken
2	Keine Trachealkanüle, ineffizientes Speichelschlucken, gelegentlich gurgelnder Stimmklang und/oder gelegentliche Expektoration (Abstände größer als 1 h) bei ausreichenden Schutzmechanismen (effektives Rachenreinigen/Hochhusten)
3	Keine Trachealkanüle, ineffizientes Speichelschlucken, häufig gurgelnder Stimmklang und/oder häufige Expektoration (Abstände kleiner oder gleich 1 h) bei ausreichenden Schutzmechanismen (effektives Rachenreinigen/Hochhusten)
4	Keine Trachealkanüle bei unzureichenden Schutzmechanismen und gelegentliches Absaugen notwendig oder Trachealkanüle dauerhaft entblockt oder Sprechkanüle/Platzhalter als Absaugmöglichkeit für Speichel
5	Trachealkanüle länger entblockt (> 12 h bis 24 h)
6	Trachealkanüle länger entblockt (> 1 h bis ≤12 h)
7	Trachealkanüle kurzzeitig entblockt (≤ 1 h)
8	Trachealkanüle dauerhaft geblockt

**Tab. 9:** Bogenhausener Dysphagiescore‑1, BODS‑2 (Kostform)

Score	Charakteristika
1	Voll oral ohne Einschränkung
2	Voll oral mit geringen Einschränkungen: Mehrere Nahrungskonsistenzen und mindestens eine Flüssigkeitskonsistenz ohne Kompensation oder Kompensation ohne Diäteinschränkung
3	Voll oral mit mäßigen Einschränkungen: Mehrere Nahrungskonsistenzen und mindestens eine Flüssigkeitskonsistenz mit Kompensation
4	Voll oral mit gravierenden Einschränkungen: Nur eine Nahrungskonsistenz und/oder eine angedickte Flüssigkeitskonsistenz mit oder ohne Kompensation
5	Überwiegend oral: Mehr als die Hälfte des Tagesbedarfs, Restbedarf via Sonde/parenteral
6	Partiell oral: Mehr als 10 TL täglich bis zur Hälfte des Tagesbedarfs, Restbedarf via Sonde/parenteral
7	Geringfügig oral: Weniger oder gleich 10 TL täglich, Restbedarf via Sonde/parenteral
8	Ausschließlich Sonde/parenteral

**Tab. 10:** Gesamtbewertung des Dysphagieschweregrads nach Bogenhausener Dysphagiescore, BODS‑1 und BODS‑2 zusammen

Summenscore	Schweregrad
2	Keine Dysphagie
3–4	Leichte Dysphagie
5–6	Mäßiggradige Dysphagie
7–10	Mittelschwere Dysphagie
11–14	Schwere Dysphagie
15–16	Schwerste Dysphagie

Der BODS‑2 (Tab. 9) bewertet die Nahrungsaufnahme.

Der Schweregrad von BODS‑1, BODS‑2 wird in einem Score von 1 (keine Störung) bis 8 (schwerste Störung) erfasst sowie in einem Summenscore aus BODS‑1 (Speichel schlucken) und BODS‑2 (Nahrung aufnehmen) bezüglich des Dysphagiegrads präzisiert, hierbei ist der Summenscore von 2 „keine Dysphagie“ entsprechend, der höchste Dysphagiegrad entspricht einem Score von 15–16 (Tab. 10).

#### Fragebogen-Assessments („assessment“: Beurteilung).

Fragebögen geben Informationen aus Patientensicht/Angehörigensicht, für die Verlaufseinschätzung und den Verlauf/das Erleben speziell der Tumorkrankheit (Beschwerden/Lebensqualität). Zu den symptomspezifischen Assessments gehören der 10 Fragen umfassende EAT-10 [18], der Sydney Swallowing Questionnaire (SSQ) [122, 134] sowie der Munich Dysphagia Test-Parkinson’s Disease (MDT-PD) [116]. Für die Einschätzung der „Lebensqualität“ werden u. a. das MD Anderson Dysphagia Inventory (MDADI) [17], der SWAL-QOL sowie Swallowing Quality of Care Questionnaire (SWAL-CARE) [78, 79] und der Dysphagia Handicap Index (DHI) [115] verwendet [modifiziert nach 4]).

#### Screeningverfahren.

Mithilfe von Screeningverfahren sollen aspirationsgefährdete Patienten entdeckt werden. Ein Goldstandard existiert bisher nicht. Aspirationsschnelltests sind allerdings wegen des geringen Zeitaufwands und geringer Kosten als Entscheidungshilfe für Sofortmaßnahmen geeignet. Stille Aspirationen werden nicht detektiert. Aspirationshinweise und Abbruchkriterien sind:

- Austrinken der gesamten Flüssigkeitsmenge nicht möglich
- Auftreten von Husten oder Erstickungsanfall bis zu 1 min nach Testende
- feucht-belegter, gurgelnder Stimmklang

Für die Dysphagie nach Schlaganfall existiert eine große Anzahl unterschiedlicher Tests. So werden u. a. der 50-ml-Wasser-Test [11], der 3‑Ounce Water Test (d. h. 90-ml-Wasser-Schluck-Test) [27], der Kidd Water Test [57], der Burke Dysphagia Screening Test [28], der Wassertest nach Daniels [24], das Bedside Swallowing Assessment (BSA) [118], das Standardized Swallowing Assessment (SSA) [93], das Massey Bedside Swallowing Screening [75], der Nishiwaki-Score [88], das Gugging Swallowing Screening [132], der Toronto Bedside Swallowing Screening Test (TOR-BSST) [74], der Acute Stroke Dysphagia Screen [36] sowie der Cough Test [133] durchgeführt (s. Zusammenfassung bei [4]). Exemplarisch bei einfacher Durchführung sind:

- 90-ml-Wasser-Test (3-Ounce Water Swallow Test)
  90 ml Wasser sollen ohne Unterbrechung aus einem Glas mit oder ohne Strohhalm getrunken werden. Vortests mit kleineren Wasserschlucken, z. B. vom Teelöffel (3 ml) können sinnvoll sein. Zeigen sich keine Symptome, liegt mit hoher Wahrscheinlichkeit auch keine Aspiration dünnflüssiger Konsistenzen vor [126].
- Gugging Swallowing Screen (GUSS)
  Die Konsistenzen „breiähnlich“/„flüssig“/„fest“ werden geprüft.

Als Hinweis auf eine Schluckstörung zählt das Auftreten von wenigstens einem der Symptome: Abschlucken nicht möglich, verzögerte Schluckinitiierung (Flüssigkeit >2 s, feste Nahrung >10 s), Drooling, unwillkürliches Husten vor/während/nach dem Schlucken bis zu 3 min, Stimmveränderungen während der Testung. Nach einer 20-Punkte-Skala wird in 4 Schweregradstufen unterteilt. Ein Protokollbogen mit Auswertung ist im Internet erhältlich [132].

#### Screening nach Tumorresektion von Kopf-Hals-Tumoren.

Für die Schluckfähigkeit nach Tumorresektion von Kopf-Hals-Tumoren werden das Frankfurter Dysphagie-Screening (Fra-DySc) [46], der Water Swallow Screening Test [47] sowie das Mann Assessment of Swallowing Ability – Cancer (MASA-C) [2, 16] verwendet (s. Übersicht in [4]).

### HNO-ärztliche Untersuchung

Zuerst erfolgt die Untersuchung der äußeren funktionellen Anatomie und des mundmotorischen Funktionskreises.

Äußerer Funktionskreis:

- Fazialisfunktion
- Drooling vorhanden?
- Lippenschluss, -spitzen, -spreizen
- Kiefer öffnen, schließen, seitlich bewegen
- Zunge herausstrecken, anheben, rückführen, kreisen, an den Lippen entlangführen, Zungenspitze in die Wangentaschen drücken
- Schnalzen
- Artikulationsdiadochokinese /pa/ta/ka/ 3‑mal hintereinander

Innerer Funktionskreis:

- Mundschleimhautbeschaffenheit, Zahnstatus (Druckstellen Prothese?)
- Defekte, Narben im Mundraum, chronische Wunden, Aufbissverletzungen
- Taktile Sensibilität im Mund durch Berührung mit Watteträger
- Beurteilung Velumbeweglichkeit durch/a/-Phonation
- Würgreflex auslösbar? (vorderes, mittleres Drittel Zunge)
- Kontraktion der Pharynxmuskulatur durch sensorische Prüfung mit Endoskopspitze

Für die Unterscheidung von peripheren und zentralen Paresen der Mundmotorik können Beobachtung und Testung Auskunft geben (Tab. 11).

**Tab. 11:** Unterscheidungsmerkmale peripherer und zentraler Bewegungsstörungen der Schluck‑/Sprechorgane

	Periphere Parese	Zentrale Parese
Willkürbewegungen	Aufgehoben	Aufgehoben oder beeinträchtigt
Reflektorische, emotionale Bewegungen	Aufgehoben	Erhalten
Muskeltonus	Erniedrigt	Anfangs erniedrigt, später erhöht
Muskelatrophie	Bei längerem Bestehen vorhanden, an Zunge ggf. Ausbildung eines Sulkus	Nicht vorhanden
Faszikulationen	Möglicherweise vorhanden	Nicht vorhanden
Hyperkinesen	Nicht vorhanden	Möglicherweise vorhanden

### Instrumentelle Untersuchungen

Unabhängig von der primär konsultierten Fachdisziplin sollte sich die Diagnostik nicht nur auf einen der genannten Bereiche beschränken, sondern funktionell hinsichtlich der Verflechtung von Pharynx und Ösophagus fachübergreifend sein [136] im Hinblick auf die Fülle der Ätiologie oropharyngealer/ösophagealer Schluckstörungen (Tab. 12).

**Tab. 12:** Interdisziplinäre Stufendiagnostik bei Dysphagie [136]

Dynamische Bildgebung	Flexible Videoendoskopie: Pharynx, Larynx, Ösophagus, Magen, Duodenum
*Radiologische Bildgebung*	
Dynamische Bildgebung	Videofluoroskopie, Hochfrequenzkinematographie, Sonographie
Statische Bildgebung	Ösophagogramm, Halsweichteile, NNH, HWS, CT, MRT, Thorax, Sonographie
*Gastroenterologische Verfahren*	Ösophageale Hochgeschwindigkeitsmanometrie, 24-h-Stunden-pH-Metrie
*Nuklearmedizinische Verfahren*	Schilddrüsen‑, Pharynx‑, Ösophagus‑, Speicheldrüsenszintigraphie
*ElektrophysiologischeVerfahren*	Pharyngeale Computermanometrie, Elektromyographie

*CT* Computertomographie,* HWS *Halswirbelsäule,* MRT *Magnetresonanztomographie,* NNH* Nasennebenhöhlen

Für die instrumentelle Schluckuntersuchung stehen speziell die Videofluoroskopie und die flexible endoskopische Evaluation des Schluckens (FEES)/flexible endoskopische Schluckuntersuchung (FESU) zur Verfügung. Beide Verfahren haben Limitationen und Vorteile. Bei Videofluoroskopien ist die Strahlenbelastung v. a. bei Kleinstkindern (< 24 Monate) häufig als nicht akzeptabel zu beurteilen, außerdem ist die befundsichere Durchführung bei Kindern entscheidend von der Erfahrung der Röntgenabteilung abhängig [137]. Videofluoroskopien stellen vorrangig dynamische Prozesse des Bolustransports dar, die intradeglutitive Aspiration ist nur durch die radiologische Untersuchung bildgebend zu beurteilen (endoskopisch nur möglich, wenn retrograde Laryngoskopie durch einen Tracheostomaschacht möglich ist).

Zur videoendoskopischen Untersuchung stehen folgende Methoden zur Verfügung:

1. Flexible (fiberoptische) endoskopische Evaluation des Schluckens, FEES (1988 Langmore et al. [63], 1991 Bastian [9], unter dem Terminus „fiberoptic [flexible] endoscopic evaluation of swallowing“ beschrieben), heute etabliert als Standarduntersuchung
2. Flexible endoskopische Schluckuntersuchung (FESU) [4]

Vorteile sind: keine Strahlenbelastung, keine besondere Patientenvorbereitung (z. B. nüchtern sein, medikamentöse Vorbereitung). Die pharyngoösophageale Phase ist gut zu beurteilen, die orale Phase ist hiervon ausgenommen. Speziell die FESU folgt keinem Untersuchungsprotokoll, sie erfolgt z. B. nach oraler Nahrungsaufnahme, um Residuen, Regurgitation in der Ruhebeobachtung und laryngeale Aktivität zu beurteilen. Die eigentliche „Schluckuntersuchung“ mit eingefärbter Nahrung erfolgt ohne endoskopische Kontrolle, da sich manche Patienten nach Einführen des Endoskops so erregen, dass eine „geordnete“ FEES nicht möglich ist.

Ziele der Schluckendoskopie sind:

- Abgrenzung struktureller und neurologischer Störungen
- Entscheidung, ob auf natürlichem Wege ausreichend Nahrung/Flüssigkeit angemessen aufgenommen werden kann oder Sonden- oder parenterale Ernährung erforderlich ist
- Entscheidung, ob Schutzmaßnahmen für die tiefen Atemwege sinnvoll sind (Tracheotomie)
- Indikation zur Speichelreduktion
- Entscheidungshilfen für Schluckversuche mit Nahrung, Nahrungsaufbau, Entblockung/Dekanülierung
- Modifikationen Nahrung/Flüssigkeit
- Empfehlung für weitere (bildgebende) Diagnostik (Videofluoroskopie, pH-Metrie, Manometrie, Gastroskopie) [136]
- Empfehlung für funktionelle Therapie
- Empfehlung zu prothetischen/operativen Maßnahmen (modifiziert nach [4])

Unmittelbar überprüfen lassen sich die Effekte von:

- Haltungsänderung von Kopf/Oberkörper
- Auswirkung von Manövern auf das Schluckvermögen

Aus dem Ergebnis der Untersuchung können weitere therapeutische Konsequenzen abgeleitet werden:

- Sondenernährung (Nasogastralsonde, PEG)
- Empfehlungen zu diätetischen Maßnahmen
- bei Erfordernis chirurgische Therapie, auch zur Schluckverbesserung (z. B. Segeldurchtrennung, Rekonstruktion aryepipglottische Falte u. a.)

Die Schluckfertigkeiten sind mit und ohne Trachealkanüle gleich [86, 97]. Tracheotomierte Kinder/Erwachsene können sowohl mit als auch ohne Kanüle, bei nasogastraler Sonde mit oder ohne untersucht werden [32]. Patienten, die eine Nasogastralsonde nicht entbehren können, sollten immer mit Sonde untersucht werden.

#### Vorbereitung einer FEES oder FESU.

Bei Kindern ist bedachtes Arbeiten wichtig. Dies gilt auch für die Untersuchung kognitiv beeinträchtigter Erwachsener, Patienten mit langer „Krankenhaushistorie“ („ich möchte keine Untersuchungen mehr“) sowie Personen im/nach Delir, Patienten mit degenerativen Krankheiten wie Demenzen. Neben Bezugspersonen kann die Anwesenheit z. B. des behandelnden Schlucktherapeuten für zusätzliche Informationen sinnvoll sein (Kinder und Erwachsene). Bei der Untersuchung von (Kleinst‑)Kindern hat sich die „Dreierregel“ bewährt (1 Untersucher, 1 Elternteil/Angehöriger, 1 assistierende Fachperson). In der Vorbereitung zur nasalen Endoskopie ist bei Kindern die Verwendung altersgerecht dosierter Nasentropfen ratsam sowie das Betupfen der Endoskopspitze (nicht der Linse) mit Gel (z. B. Xylocain-Gel). Der nasal-endoskopische Transfer ist für Untersucher und Kind einfacher, die Akzeptanz höher. Die Schleimhautanästhesie der vorderen Nasenabschnitte beeinflusst die Schluckfähigkeit nicht [52]. Die Applikation von anästhesierendem Spray in die Nase ist schmerzhaft und vermindert häufig schlagartig die Mitarbeit des Kindes, auch werden Schleimhautareale zu großflächig anästhesiert. Die Vertrauensperson, meistens ein Elternteil, nimmt das Kind auf den Schoß (auch ältere dürfen auf dem Schoß sitzen, wenn sie wollen), die Arme des Kindes werden leicht am Körper gehalten. Die Konsistenzen entsprechen Entwicklungsalter, Vorlieben und individuellen Fragestellungen. Erwachsene werden im Untersuchungsstuhl aufrecht hingesetzt oder können im Rollstuhl untersucht werden. Wichtig ist die Durchführung der FEES unter Alltagsbedingungen, eine erzwungene aufrechte Körperhaltung, wenn der Patient sonst nur im Bett liegt, ergibt keine aussagefähigen Befunde für die Alltagssituation, zumal auch die Oberkörperlage von 40 % gute Voraussetzungen für eine Schluckuntersuchung bietet.

#### Vorteile der FEES, FESU.

- Strukturen und Einzelfunktionen beurteilbar, die radiologisch nicht/unzureichend zu erfassen sind, z. B. Entzündungen, Defekte, diskrete Bewegungsstörungen
- Aspirationen von Speichel, die beim Schlucken von Fremdsubstanzen, z. B. Kontrastmittel, nicht in Erscheinung treten [90]
- Wiederholbar, auch zur Therapieevaluation
- Keine Strahlenexposition
- Die Untersuchung ist „vor Ort“, z. B. Intensivstationen, Alten‑/Pflegeheime
- Nutzung als Biofeedback-Verfahren
- Geringere Kosten als radiologische Diagnostik

#### Nachteile der FEES, FESU.

- Orale Phase nicht zu beurteilen
- Während pharyngealer Phase Zungengrund, Rachen, Kehlkopfstrukturen nur unmittelbar vor/nach pharyngealer Phase zu sehen, „Mitnahmeeffekt“ des sich hebenden Velums und des sich kontrahierenden Pharynx meist in Kontakt mit der Rachenhinterwand, „white out“ [84] behindert die ausreichende Beurteilung der entscheidenden Komponenten Kehlkopfverschluss und Öffnung oÖS
- Beurteilung Ösophagusfunktionen eingeschränkt, bei mangelhafter Entfaltung Beurteilung Schleimhautrelief unzureichend, Endoskope üblicherweise zu kurz, um unteren Ösophagussphinkter einsehen zu können
- Menge aspirierten Materials nicht sicher einzuschätzen, wenn kein Tracheostoma vorliegt zur Trachea‑/Bronchusabgangsbeurteilung
- Bei motorischen Störungen wie Paresen, Ataxie, Tremor oder Dystonie, deutlich längsovalem Pharynxlumen oder ausgeprägter Larynxschiefe schwierige Positionierung des Endoskops ohne Torquierung, laterale Wegdrängung
- Vasovagale Reflexe mit Hypotonie/Bradykardie, Kollapsneigung, z. B. bei Berührung der Epiglottis durch Endoskopspitze, oder laryngealer Spasmus bei Berührung der Taschenfalten/Stimmlippen, aber selten [6].

Absaugmöglichkeit, Kreislaufbehandlung, Notfallkoffer, ggf. eine liegende Venenverweilkanüle sowie die Möglichkeit einer Notfallbronchoskopie sind für eine sichere Durchführung der flexiblen endoskopischen Schluckuntersuchung empfehlenswert [4, 5].

Sinnvolle Materialien zur FEES-Durchführung sind [4]:

- Wasserglas (alternativ Pappbecher), Löffel, Strohhalm, Holzspatel
- Götterspeise
- Flüssigkeit: Wasser, gefärbt mit blauer Lebensmittelfarbe
- Andickungsmittel zum Variieren der Flüssigkeit
- Feste Substanzen: Zwieback
- Reihenfolge variabel, jedoch üblicherweise Götterspeise, Wasser, Zwieback

Milchprodukte sind ungeeignet, unabhängig von ihrem Milchsäure- und Milchzuckeranteil, denn sie verschmieren häufig die Linse, die sich durch „Abwischen an der Pharynxmukosa“ häufig nicht mehr reinigen lässt.

#### FEES-Durchführung.

Auf Anfrage verwendet man das Nasenloch, durch das der Patient besser atmen kann. Bei kleinen Kindern oder allgemein unruhigen Patienten favorisiert man die Seite, die man selbst bevorzugt. Bei Nasogastralsonden kann es sinnvoll sein, sich entweder an der Sonde entlang zu schieben, bei wenig Platz (Säuglinge, Kleinstkinder) ist die Gegenseite wegen Platzmangels keine Alternative.

Positionierung des Endoskops:

- Position 0; Nasenrachen: Tubeneingänge und Gaumensegel. Durch /k/-Phonation („kalter Kaffee“) Beurteilung Velumokklusion getrennt für beide Seiten
- Position 1: Höhe der Gaumenbögen, Pharynx und Larynx mit Epiglottis, Panoramaübersicht
- Position 2: hinter oberem Epiglottisrand, Petiolus und Larynx sowie Hypopharynxeingang beidseits mit Pharynxrückwand, subglottische Region
- Position 3: postkrikoidal, Pharynxrückwand Arytänoide/Interarytänoidregion, Sinus piriformis bds. [4].

Die Lage der Endoskopspitze ist während der Schluckuntersuchung optimal auf Position 1, um die laryngealen Bewegungen nicht zu beeinträchtigen [13] und um Boluspenetrationen/Aspirationen in Glottis und Trachea nachzuweisen (**Cave**: vasovagaler Reflex). Um den oÖS zu passieren, wird die Endoskopspitze oberhalb des ipsilateralen Sinus piriformis (Seite des Endoskops in der Nase) platziert. Beim Schlucken wird dann das flexible Endoskop vorsichtig durch den oÖS vorgeschoben und dessen Funktion beobachtet. Das Trinken von Wasser mit dem Strohhalm führt zu einer physiologischen Hypästhesie, der Transfer in den Ösophagus gelingt so leichter.

Die Endoskopie beginnt ohne Nahrung mit Blick auf Pharynx und Larynx und zur Erfassung von Residuen/Penetration/Aspiration von Speichel oder Sekret (Tab. 13).

**Tab. 13:** Beurteilungsparameter vor Schluckversuch

Untersuchung	Befunde
Ruhebeobachtung	Strukturveränderungen: Defizite, Ödeme, Narben, Vorwölbungen der Rachenhinterwand, Laryngitis, Laryngitis posterior
	Stimmlippenstellung zur Glottisachse, Tonus-(Form‑)Veränderungen
	Stimmlippenparesen, Hyperkinesen (Myoklonien, Tremor) Larynxskelett
	Zeichen des gestörten Schluckablaufs: Residuen von Speichel/Sekret und Penetration, Aspiration von Speichel/Sekret (reflektorisches Husten, spontanes Räuspern)
Phonation	Phonation /e/ kurz hintereinander: Beurteilung der diadochokinetischen Beweglichkeit (bei spastischer Parese vermindert)

Patienten ohne/mit bisheriger oraler Nahrungszufuhr erhalten zunächst Götterspeise(etwa 3–5 g = 1 Teelöffel). Die halbfeste Konsistenz ist meistens leichter zu schlucken als Flüssigkeiten und feste Konsistenzen. Götterspeise ist gleitfähig und verflüssigt sich bei Erwärmung im Körper. Sie kann bei Aspiration leicht abgehustet bzw. abgesaugt werden oder wird ohne größere Reizerscheinungen resorbiert. Die grüne Farbe eignet sich zur Beurteilung von Residuen oder Epipharynxregurgitation. Für jede Konsistenz wird Boluskontrolle, Triggerung Schluckreflex, Larynx-Hyoidelevation und Leaking beurteilt. Eine reduzierte pharyngeale Schluckaktivität zeigt verzögerte Anterior- und Aufwärtsbewegungen der Aryknorpel [1, 25], eine verzögerte Anhebung des Zungengrunds. Beim Bolusabgleiten in die Valleculae bzw. die Recessus piriformes hebt sich der Kehlkopf spät an ohne Epiglottisneigung nach dorsal [13, 25], bei einer verlangsamten „Freezed-Rückstellung“ der Epiglottis sieht man häufig ein endoskopisches Zurückspringen der Epiglottis, z. B. auch durch Inkarzerierung an der Rachenhinterwand durch Spondylophyten. Nach der Bolusgabe und erster Schluckbewegung können *Bolusresiduen* an den Pharynxwänden, in den Valleculae und den Recessus piriformes, in der Postkrikoidregion mit/ohne Versuch des Rachenreinigens beurteilt werden, *Penetrationen* im Larynxeingang auf laryngealer Epiglottisfläche, über den aryepiglottischen Falten, über der Interarytänoidregion ohne/mit Auslösung des Hustenreflexes, *Aspiration*en in die Glottis, in die subglottische Region ohne/mit Auslösung eines Hustenreflexes. Die Tab. 14 fasst die Nomenklatur pathologisch-endoskopischer Befunde zusammen.

**Tab. 14:** Übersicht zur Nomenklatur (endoskopisch) bei Bolustransportstörungen [4]

Drooling	Herauslaufen Bolus oder Speichel aus Mund
Regurgitation	Hochwürgen von Nahrung
Leaking	Abgleiten Bolus in Rachen vor Auslösung Schluckreflex aufgrund gestörter Oralmotorik mit beeinträchtigter Boluskontrolle
Pooling	Ansammlung von Substanzen im Hypopharynx aufgrund fehlender oder verspäteter Auslösung Schluckreflex
Residuen oder Retentionen	Ansammlungen von Speichel, Sekret, Bolusanteilen in Mundhöhle, an Hypopharynxwänden, in Valleculae, Recessus piriformes, Postkrikoidregion
Penetration	Übertritt von Speichel, Sekret, Bolusanteilen in Kehlkopftrichter ohne Durchtritt durch Stimmlippenebene
Aspiration	Durchtritt von Speichel, Sekret, Bolusanteilen durch Stimmlippenebene hindurch
Nasale Regurgitation/Penetration	Übertritt von Speichel, Sekret, Bolusanteilen in Nasopharynx, die Nasenhaupthöhle oder aus der Nase

Die Tab. 15 zeigt eine Zusammenstellung endoskopischer Befunde mit ihrem Störungsbild.

**Tab. 15:** Laryngoskopische Befunde: pathophysiologisches Korrelat [108]

Endoskopischer Befund	Pathophysiologisches Korrelat
Leaking (vorzeitiger Übertritt) prädeglutitive Aspiration prädeglutitives Pooling	Gestörte orale Boluskontrolle
	Verspätete Reflextriggerung
*Residuen*	
Hinterer Nasengang	Ungenügender velopharyngealer Verschluss
Pharynxwände	Ungenügende Pharynxkontraktion
Valleculae und Recessus piriformis	Ungenügende Zungenschubkraft
	Reduzierte Kehlkopfhebung
	Reduzierte Zungenbasisretraktion
	Reduzierte Pharynxkontraktion
Postkrikoidregion	Reduzierte Öffnung des oberen Ösophagussphinkters
	Reduzierte Kehlkopfhebung und -anteriorbewegung
*Penetration*	
Laryngeale Epiglottisfläche aryepiglottische Falten Taschenfalten Oberfläche der Stimmlippen	Reduzierte Kehlkopfhebung
	Reduzierte Dorsalbewegung der Epiglottis
	Reduzierte Adduktion des Aditus laryngis
	Sensibilitätsstörung, Störung der Schluckreflexauslösung
*Aspiration*	Reduzierter oder nicht zeitgerechter Verschluss des Aditus laryngis inklusive verminderter Epiglottiskippung
	Reduzierter oder nicht zeitgerechter Glottisschluss
	Sensibilitätsstörung; Störung der Schluckreflexauslösung, der Hustenreflexauslösung

Für Empfehlungen sind Bewertungen von Aspirationszeichen in der klinischen, endoskopischen und/oder röntgenologischen Diagnostik erforderlich. Verschiedene Einteilungen sind erarbeitet worden, die allerdings unterschiedliche Untersuchungsmodalitäten (klinische Beurteilung, Videoendoskopie, Videofluoroskopie) und verschiedene Kriterien bewerten (Auffälligkeiten bei der klinischen Untersuchung einschließlich dysarthrischer Symptome, Residuen, Aspirationssymptome von Speichel und/oder Nahrung, Fähigkeit zur Nahrungsaufnahme) [4]. Die 8‑stufige Einteilung von Rosenbek et al. 1996 [101] wurde für den radiologischen Befund und für die endoskopische Untersuchung evaluiert [99]. Sie hat sich für den Klinikgebrauch und zur Bewertung von Studien als geeignet erwiesen. Die deutsche Version wurde 2014 validiert [48]. Sie bewertet im Wesentlichen das Vorhandensein und die Reaktion auf „Material“ (den Bolus) im Kehlkopf/in der Trachea (Tab. 16). Das Ausmaß der Residuen als Zeichen mangelnder Schluckeffektivität wird nicht berücksichtigt.

**Tab. 16:** Deutsche Version der Penetrations-Aspirations-Skala nach Rosenbek [48]

1	Material dringt nicht in den Luftweg ein
2	Material dringt in den Luftweg ein, verbleibt oberhalb der Stimmlippen und wird aus dem Luftweg ausgestoßen^a^
3	Material dringt in den Luftweg ein, verbleibt oberhalb der Stimmlippen und wird nicht aus dem Luftweg ausgestoßen^a^
4	Material dringt in den Luftweg ein, kontaktiert die Stimmlippen und wird aus dem Luftweg ausgestoßen
5	Material dringt in den Luftweg ein, kontaktiert die Stimmlippen und wird nicht aus dem Luftweg ausgestoßen
6	Material dringt in den Luftweg ein, passiert bis unter die Stimmlippen und wird in den Larynx hinein oder aus dem Luftweg ausgestoßen
7	Material dringt in den Luftweg ein, passiert bis unter die Stimmlippen und wird nicht aus der Trachea ausgestoßen, trotz Bemühung
8	Material dringt in den Luftweg ein, passiert bis unter die Stimmlippen, und es wird keine Bemühung zum Ausstoßen unternommen

^a^Ausstoßen inkludiert: Husten, Räuspern und Schlucken (nach Rücksprache mit J. Rosenbek)

Die Sekretbeurteilungsskala nach Murray et al. [84] wurde ebenfalls in der deutschen Übersetzung validiert und existiert in einer Lang- und Kurzversion ([95]; s. in [4]). Die Tab. 17 zeigt die Schweregradeinteilung einer Aspiration nach dem laryngoskopischen Befund.

**Tab. 17:** Schweregradeinteilung der Aspiration nach dem laryngoskopischen Befund

Schweregrad	Aspirationssymptomatik
0	Keine Aspiration
I	Gelegentliche Aspiration bei erhaltenem Hustenreflex
II	Permanente Aspiration bei erhaltenem Hustenreflex oder gelegentliche Aspiration ohne Hustenreflex mit gutem willkürlichem Abhusten
III	Permanente Aspiration ohne Hustenreflex mit gutem willkürlichem Abhusten
IV	Permanente Aspiration ohne Hustenreflex, ohne willkürliches effektives Abhusten

Bei Schweregrad I und II können konservative Maßnahmen (Diätanpassung, funktionelle Schlucktherapie, Refluxprophylaxe) evtl. ausreichen. Bei Schweregrad III müssen weitreichendere Entscheidungen erwogen werden, vom Verbot oraler Nahrungszufuhr bis zu Tracheotomie und geblockter Kanüle. Mit dieser Einteilung werden das Vorliegen eines Hustenreflexes und die Fähigkeit zum willkürlichen Abhusten erfasst.

Da die Endoskopie nicht den gesamten Schluckvorgang und nur einen Teil der Ursachen der Störungen definieren kann, sollte eine Videofluoroskopie in Erwägung gezogen werden.

Nach Einführung der Hochgeschwindigkeitskinematographie in die Analyse des Schluckvorgangs [44] galt die radiologische Untersuchung zunächst viele Jahre als Goldstandard [71]. Bei optimaler Erfassung des kompletten Schluckablaufs ist sie jedoch technisch aufwendig, mit Strahlenbelastung verbunden und nur bei kooperativen Patienten durchführbar. Nach dem radiologischen Befund kann der Schweregrad der Aspiration nach Hannig ([44]; Tab. 18) eingeteilt werden, es handelt es sich um ein geschätztes quantitatives Volumen, das allerdings im Alltag bei minimalen Veränderungen in Verlaufskontrollen um die „10%-Marke“ Unsicherheiten birgt. Der von Farneti 2008 vorgestellte „Pooling Score“ [38] beurteilt sowohl Residuen als auch Ansammlungen in Kehlkopfeingang und Glottis (also eigentlich Penetration und Aspiration) nach Lokalisation, Menge und „Management“.

**Tab. 18:** Einteilung radiologisch Aspirationsgrade nach Hannig [44]

Schweregrad	Aspiration	Hustenreflex
1	Aspiration des im Aditus und Ventriculus laryngis retinierten Materials	Erhalten
2	Aspirationsvolumen von etwa 10 % des Bolus	Erhalten
3	Aspiration ≤10 % des Bolus Aspiration >10 % des Bolus	Reduziert erhalten
	Aspiration >10 % des Bolus	Fehlt

Zusammenfassend gibt es umfassende Diagnostikinstrumente, die es dem HNO-Arzt und Phoniater ermöglicht, selbst diagnostisch tätig zu werden und seine Expertise umfassend einzusetzen. Es liegt also durch eigene Erfahrung, die Verwendung verschiedener Assessments, Fragebögen und letztendlich die FEES/FESU in der eigenen Hand, in das diagnostische Geschehen einzugreifen und durch diese Techniken die eigene Verantwortung gegenüber dem Patienten zu erweitern.

Letztendlich steht hinter dieser Diagnostik die umsichtige und kluge Überlegung, nicht allein im Sinne der fachlichen Expertise über Maßnahme oder Empfehlungen zu entscheiden. Ziel ist es eher, Konzepte nicht nur nach kategorischen Ansichten („Sie bringen den Patienten um, wenn Sie ihm was geben“), sondern in einem Kontinuum von Möglichkeiten der Lebens‑/Alltagsqualität, der ethischen Grundsätze zu erarbeiten.

## Konservative Therapieoptionen

### Funktionelle Therapie

Die konservative Dysphagiebehandlung umfasst konservativ-übende, medikamentöse, elektrostimulatorische Verfahren [107]. Die funktionelle Therapie aus dem konservativ-übenden Spektrum beruht auf einer funktions- und problemorientierten Vorgehensweise, der Normalisierung von gestörten Schluckmustern, die sich ein 3 Bereiche einteilen lässt: Kompensationsverfahren, restituierende Verfahren und adaptive Verfahren [11].

#### Kompensatorische Strategien.

Kompensatorische Maßnahmen beinhalten Haltungsänderungen während des Schluckens, die verschiedenen Schluckmanöver und die pharyngealen/glottalen Reinigungstechniken mit dem Ziel der verbesserten Schluckeffizienz, der Aspirationsvermeidung.

- Ess‑/Trink-*Begleitung*: persönliche Hilfestellungen/Supervision während des Essens kann die Automatisierung veränderter Körperhaltungen beim Essen unterstützen sowie ggf. eine manuelle Unterstützung beim Anreichen von Nahrung sowie Training von Schlucktechniken usw. [11]
- Ess‑/Trink-*Gewohnheiten*:
  - Veränderungen Essverhalten, z. B. ausreichend Zeit lassen (ggf. Pausen einlegen), beim Essen nicht reden, kleine Boli zu sich nehmen, gut kauen (ggf. Nachschlucken), optimale Körperhaltung, Mischkonsistenzen vermeiden [4]
- Differenzierte Schluckmanöver:
  - Kopfneigung nach vorne („chin tuck“): Beim Schlucken Kopfneigung nach vorn-unten (Kinn in Richtung Brust). Förderung Zungenbeinelevation, Verminderung Penetration, besonders bei Flüssigkeiten, Effekte vom Schweregrad der Schluckstörung abhängig: je ausgeprägter die Störung, desto weniger wirksam ist es [104]
  - Kopfneigung zur stärkeren Seite: bei einseitiger oraler und/oder pharyngealer Parese Bolusleitung über die „stärkere“ Seite leiten [11]
  - Kopfdrehung entweder zur stärkeren oder betroffenen Seite: dadurch Bolustransport auf andere Seite geleitet, Öffnung des oÖS erleichtert, Kippen der Epiglottis deutlich beschleunigt, nachfolgend verbesserter Transport und Aspirationsschutz [60]
- Schlucktechniken
  - Kräftiges Schlucken: Aufforderung, so kräftig wie möglich zu schlucken. Studien zeigten einen erhöhten pharyngealen Druck und verbesserten Transport [11], obwohl die Evidenz zur Effektivität gering ist [7]
  - Supraglottisches Schlucken: Aufforderung, den Atem anzuhalten vor und während des Schluckens, um Glottisschluss zu erreichen. Nach dem Schlucken sofortiges Räuspern und Nachschlucken [30]
  - Mendelsohn-Manöver: Aufforderung, beim Schlucken Larynxelevation zu halten, dadurch Schluckprozessverlängerung, Hyoidelevation erhöht, Kontraktion Pharynxmuskulatur verstärkt [76]
  - Diätische Maßnahmen: Veränderung der Konsistenz (z. B. durch Pürieren) [103]
  - Eindicken von Flüssigkeiten umstritten. Zwar wurden geringere Aspirationsereignisse beim Eindicken von Flüssigkeiten festgestellt [120], das Eindicken führt jedoch zu einer signifikant geringeren Flüssigkeitsaufnahme [77]. Hansen postuliert, dass es bisher keine überzeugenden Daten gibt, dass das Eindicken die Rate der Pneumonien signifikant vermindert, die Mortalität verbessert [45]. Im Einzelfall sind Eindickungsmittel immer dann empfehlenswert, wenn sie nach Einschätzung der Betroffenen ein störungsfreieres Schlucken ermöglichen oder der Stress beim Trinken reduziert wird
  - Trink/Esshilfen: spezielles Geschirr/Besteck kann das selbständige Essen erleichtern, das geeignete Platzieren des Bolus ermöglichen (z. B. auf die hintere Zunge) [11]
  - Prothesen: z. B. Obturatorprothesen bei Velumdefekten oder Zungenparesen zur Verbesserung Zungen-Gaumen-Kontakt [140]

Daneben gibt es ganzheitliche Konzepte mit neurophysiologischem Ansatz, z. B. die Facio-orale Trakt-Therapie F.O.T.T.®, die propriozeptive neuromuskuläre Fazilitation (PNF) sowie die Orofaziale Regulationstherapie nach Castillo Morales (ORT) [11], die durch die Förderung einer ganzkörperlichen Eutonie eine Normotonisierung der motorischen Schluckmuster durch sensorische und mechanische Stimulationen erreichen soll. Die Behandlungskonzepte sind bezüglich ihrer Wirksamkeit nicht wissenschaftlich untersucht [30].

#### Restituierende Strategien.

Das Ziel besteht in einer Normalisierung von gestörten Schluckabläufen durch Training zentraler Innervierungsmuster, z. B. durch externe Stimulation.

- Sensorisch-thermale Stimulation: eisgekühlte Thermosonden zur Berührung der Gaumenbögen [25], alternativ Zerkauen einzelner Eispartikel, die die Basis der vorderen Gaumenbögen umspülten Danach Aufforderung zum Schlucken. Evidenz zur Effektivität sehr eingeschränkt [109]
- Übungen:
- Masako-Manöver: Zungenspitze wird während des Schluckens von Speichel zwischen die Zähne „geklemmt“ und dadurch stabilisiert. Diese Übung erhöht und verlängert Kontraktion der oberen Rachenmuskeln und dadurch einen verbesserten Zungenbasis-Rachen-Verschluss [94]
- Shaker-Manöver (auch „chin tuck against resistance“): Kinn wird Richtung Brust gedrückt entweder gegen die Schwerkraft (Shaker Manöver) oder gegen einen Widerstand (z. B. Ball, gerolltes Handtuch oder spezielles Gerät, „chin-tuck against resistance“). Verbesserung Stärke/Ausdauer suprahyoidale Muskulatur und Öffnung des oÖS [70, 117]
- Zungenmuskeltraining: Die am häufigsten untersuchte Zungenübung ist die Zunge-gegen-Gaumen-Übung: Aufforderung, Zunge gegen harten Gaumen zu drücken, halten und loszulassen, Bewegung etwa 10-mal zu wiederholen, Zeitspanne über etwa 8 Wochen. Training Zungendruckverstärkung/oraler Transport [59]
- Mendelsohn-Manöver als Leerschluckübung. Die genannte Schlucktechnik kann auch restituierend eingesetzt werden: durch Wiederholung dieses Manövers Verbesserung der Dauer der Zungenbeinelevation und Dauer der oÖS-Öffnung [76]

Die funktionelle Therapie sollte früh beginnen, insbesondere um die Oralisierung zu unterstützen, um narbig-rigide Veränderungen der für das Schlucken wichtigen Strukturen zu reduzieren und um bisher nichtpraktizierte Schluckhaltungsmuster zu automatisieren. Mit Abschluss der Behandlung bietet sich der Beginn einer Therapie nach spätestens 6–7 Wochen an, aber auch früher, wenn sich der Patient hierzu in der Lage fühlt. Insgesamt ist es psychosozial wichtig, die Motivation und Hoffnung auf eine Verbesserung der Schluckfertigkeiten frühzeitig zu unterstützen.

### Respiratorische Übungen

Respiratorische Übungen beinhalten sämtliche Verfahren, die die Exspirationskraft und pulmonale Kapazitätserhöhung zum Ziel haben.

- Training exspiratorischer Muskulatur („expiratory muscle strength training“, EMST): Ausatmung gegen erhöhten Widerstand (z. B. mit Atemtrainer). Training Atemmuskulatur und Kräftigung submentaler Muskulatur mit positiver Auswirkung auf die Schluckeffizienz [21]
- Schluck-Atmen-Koordination: relativ neuer Einsatz; durch gezielte Übungen Koordinationsverbesserung, nachfolgend reduziertes Aspirationsrisiko [72]

### Pharmakotherapie

Die Pharmakotherapie soll Neurorezeptoren, die für das Schlucken relevant sind, entweder aktivieren/sensibilisieren und/oder dystone hyperaktive Muskelaktivitäten reduzieren.

- TRPV1-Agonisten (Transiente Rezeptor-Potenzial-Kationenkanäle der Unterfamilie V) z. B. Capsaicinoide und Piperine (schwarzer Pfeffer), Substanzen wirken auf TRPV1-Rezeptoren im Oropharynx mit Schluckreflexlatenzverkürzung, Larynxelevationsverbesserung, Effekte nur während der Substanzgabe nachweisbar, nicht langfristig auch ohne Gabe [19]
- Dopaminergika (L-Dopa, Amantadin, Cabergolin) als Neurotransmitter können auch Dysphagien beim Parkinsonsyndrom verbessern [8]. Metoclopramid hat auch bei einigen Studien positive Wirkungen auf neurogene Schluckstörungen gezeigt [19].
- ACE-Hemmer: Nebenwirkung der ACE-Hemmer erhöhte Auslösbarkeit des Husten‑/Räusperzwangs, kann therapeutisch eingesetzt werden, um die Latenz des Schluckreflexes zu verkürzen, die unwillkürliche Schluckfrequenz zu erhöhen und das Risiko für nächtliche Aspirationen zu verringern [3, 5]. Effekte diesbezüglich sind widersprüchlich [8]. Kalziumkanal-Antagonisten (Nifedipin) können Effekte bei Dysphagie zeigen [20].
- Botulinumtoxine (BTX): bei symptomatischen lokalen Dystonien im Hals‑/Kopfbereich (z. B. oromandibuläre Dystonie, krikopharyngeale Dystonie, retropharyngeale Störung). Wirkprinzip ist eine chemische Denervierung an der motorischen Endplatte. Bei Patienten mit Torticollis spasticus stellt die BTX-Behandlung ein bewährtes Konzept da, das Risiko einer Dysphagie durch Diffusion des Toxins in die larynxnahe Peripherie ist vorhanden, wenn auch reversibel [65]. Botulinumtoxininjektion zur Speichelexkretionsreduktion in die großen Speicheldrüsen, alle 3–4 Monate Wiederholung erforderlich [53]
- Hypersalivation: anticholinerge Muscarinrezeptor-Antagonisten (MRA), z. B. Atropin, Scopolamin und Glycopyrrolat: intravenös/intramuskulär, transdermal (Pflaster) oder per Tropfen/Spray, Scopoderm und Atropin „off-label use“ (Glycopyrrolat 2016 bei Kindern ab 3 Jahren und Jugendlichen zugelassen) [29, 30]

Wegen der niedrigen Anzahl an Studien, Daten und teilweise widersprüchlichen Ergebnissen sind pharmakologische Behandlungen (außer BTX) nicht zu empfehlen [20].

### Neurostimulation

Nichtinvasive (ohne Nadelelektroden) Neurostimulationen können in zentral oder peripher wirksame Stimulationen eingeteilt werden:

- Zentrale Stimulation:
- Transkranielle Gleichstromstimulation (tDCS): durch Elektrostimulation über Elektroden an der Hautoberfläche des Schädels wird die Schwelle zur Entladung stimulierter Neuronen reduziert, die Entladungsrate moduliert
- Transkranielle Magnetstimulation (TMS): durch ein magnetisches Feld mit aufgelegten Spulen werden Aktionspotenziale der Nervenzellen stimuliert oder gehemmt, abhängig von der Impulsquote [20]
- Periphere Stimulation:
- Transkutane neuromuskuläre Elektrostimulation (NMES): Durch Elektroden an der Hauptoberfläche am Hals werden während des Schluckens (bzw. der Schlucktherapie) gezielt Nerven und Muskelgruppen gereizt. Bis jetzt gibt es keinen eindeutigen Hinweis auf eine Effektivität dieses Verfahrens [128]
- Pharyngeale Elektrostimulation (PES): durch transkutane elektrische Stimulation der Pharynxmukosa (mit einer Sonde transnasal oder transoral) neuroplastische Veränderungen retrograd fördern [20]

Prinzipiell können diese Verfahren vielversprechend sein, ihr kurz- und langfristiger Effekt bleibt abzuwarten [30].

### Radiatio bei Hypersalivation

Die Bestrahlung der großen Speicheldrüsen kann zu einer langfristigen Reduktion der Speichelproduktion führen. Allerdings müssen Nebenwirkungen und Strahlenbelastung sowie langfristiges onkologisches Risiko insbesondere bei Kindern und Jugendlichen berücksichtigt werden [67].

## Chirurgische Therapieoptionen

Chirurgische Eingriffe bei Dysphagien sind indiziert, wenn konservative Maßnahmen nicht helfen oder aufgrund einer Vorbehandlung eine wegweisende Verbesserung (z. B. Vernarbung, Rigidität Gewebe nach onkologischer Operation, Trauma [z. B. Interaryfibrose nach multiplen Intubationen]) zeitlich nicht mehr zu erwarten ist.

Chirurgische Eingriffe können in 2 Gruppen eingeteilt werden, je nachdem, welche funktionelle Verbesserung sie erreichen sollen.

- Verbesserung der organisch und/oder neuromuskulär verursachten Bolustransportstörung
- Verbesserung der organisch und/oder neuromuskulär verursachten Aspiration

### Verbesserung Bolustransport.

- Positionierung der Laryngohyoid-Einheit durch Raffung, Fixierung
- Protektion des Larynxeingangs durch Absenkung Epiglottis/Zungengrundkomplex oder Elevationsplastik der Interarytänoidregion
- Öffnung oberer Speiseröhrensphinkter durch Bougierung oder Myotomie:
- Eine Myotomie kann entweder extern (laparoskopisch) oder endoskopisch (minimalinvasiv) durchgeführt werden zur Verbesserung der bei Öffnung des oÖS in der pharyngealen Phase. Die Myotomie ist am effektivsten, gefolgt von Dilatation, nachfolgend die Botulinumtoxin-Injektion (wenn auch am wenigsten invasiv) [15]
- Bei zervikalen Osteophyten operative Abtragung möglich, wenn eindeutig mechanische Obstruktion besteht, eine Verbesserung der pharyngealen Schluckkompetenz ist nicht garantiert [25]
- Ossifikationen Lig. stylohyoideum (Eagle-Syndrom), Elevation der Laryngohyoid-Einheit kann behindert sein und Odynophagie auslösen: Resektion des Styloids kann sinnvoll sein [4]
- Hyperplastischer Zungengrund, Epiglottiszysten: klinisch bestehen postoperativ weiterhin oft Schluckschwierigkeiten. Epiglottiszysten sind selten Ursache einer Dysphagie [4]
- Aussackungen im muskelfreien Dreieck der Membrana thyrohyoidea: Ist eine Vagusparese ausgeschlossen, kann körpereigener Knorpel die Wand verstärken, um die Aussackung zu reduzieren [4]
- Zenker-Divertikel: endoskopische oder offene Operation
- Unilaterale Pharynxwand-Defekte: Bei Vagusparalyse kann die pharyngeale Wand sich sackartig aufweiten. Eine Pharynxteilresektion/-straffung kann eine funktionelle Integrität herstellen [4]
- Segelbildung: alle narbigen Veränderungen im Pharynx (z. B. aryepiglottische Falte) und im Ösophagus können die Boluspassage verhindern, eine Durchtrennung und ggf. Mukosaläppchenbildung zur Vermeidung eines erneuten Narbensegels können sinnvoll sein [4]
- Verbesserung velopharyngealer Verschluss
  - Annäherung Velum an Rachenhinterwand durch Velopalatoplastik, ggf. mit Unterfütterung Velumhinterwand durch körpereigenes Fett oder Silikon
- Verbesserung laryngealer Verschluss
  - Stimmlippenaugmentation bei Stimmlippenparese (einseitig), Stimmlippenschluss zum Schutz der Atemwege beim Schlucken
  - Ödemabtragung z. B. bei chronischen Arytänoidödemen

### Verbesserter Aspirationsschutz.

- Medianverlagerungstechniken der Stimmlippe
  - Endoskopische Injektions- oder Implantationsaugmentation durch Einsatz von körpereigenem oder -fremden Material zur Rekonstruktion der Stimmlippe
  - Thyroplastik: durch ein Schildknorpelfenster wird entweder körpereigenes oder körperfremdes Material eingebracht, um eine Verlagerung der gelähmten Stimmlippe und dadurch einen verbesserten Glottisschluss herzustellen
  - Arytänoid-Adduktion (meist mit Thyroplastik kombiniert): der gelähmte Aryknorpel wird an Proc. muscularis gezogen und ventral am Knorpelfenster befestigt, um paretische Stimmlippe in medialere Stellung zu bringen [50]
  - Augmentationen Taschenfalte(n) mit Fett
  - Epiglottisabsenkung operativ nach dorsal verlagert für besseren Schutz Larynxeingang
  - Elevationsplastik Interarytänoidregion: Bei einer Interarytänoid-Aspiration kann chirurgisch diese „Schwelle“ erhöht werden [4]
  - Positionierung Laryngohyoid-Einheit: Verlagerung Laryngohyoid-Einheit nach ventral durch Elevation Epiglottis, auch mit Myotomie M. cricopharyngeus kombinierbar [4]
- Spezielle Chirurgie bei lebensbedrohender Aspiration
  Als Ultima ratio ist ein temporärer oder dauerhafter Verschluss des Larynx möglich, das bedeutet die Trennung der Luft- und Schluckwege. Dieses Vorgehen ist immer mit einer dauerhaften Tracheotomie verbunden
  - Supraglottischer Verschluss Kehlkopf: Die paarigen Hypopharynxanteile werden zusammengenäht (z. B. Taschenfalten-Vernähung und Epiglottoaryepiglottopexie). Inwiefern diese Operation reversibel ist, bleibt kritisch abzuwarten durch Vernarbungs- und Umstrukturierungsprozesse
  - Laryngotracheale Separation/Diversion: Tracheadurchtrennung, der untere Trachealstumpf wird Tracheastoma, der obere Trachealstumpf entweder zugenäht (Separation) oder in Ösophagus umgeleitet (Diversion), Reversibilität ist nicht garantiert [4]
  - Reversibler Trachealverschluss mit Laryngohyoidopexie durch Einsetzen Haut-Platysma-Faszien-Insellappen und Laryngohyoidopexie mit subglottischem plastischem Verschluss Atemweg. Einsetzen eines Stimmventils möglich [4]
  - Laryngektomie: permanente Trennung Luft‑/Speisewege. Trotzdem sind nicht alle Patienten danach in der Lage, sich ausreichend oral zu ernähren [24]. Dieser Eingriff wirft insbesondere bei schwerstbetroffenen Kindern ethische Fragen auf. Letztendlich muss zwischen eigenen ethischen Vorstellungen und dem Wunsch auch der Angehörigen abgewogen werden

Die Dysphagie mit Speichelaspiration kann ein Grund sein, ein Tracheostoma anzulegen, entweder als perkutane Dilatations- oder als chirurgische Tracheotomie [26], wobei auf Intensivstationen die Dilatationstracheotomie häufiger eingesetzt wird. Kontraindikationen gegen die Punktionstracheotomie nach Bause [10] sind eine akute Ateminsuffizienz (Notfallsituation), nicht korrigierbare kombinierte Gerinnungsstörungen sowie die hochgradige Kreislaufinstabilität. Eine frühe Tracheotomie kann sinnvoll sein (innerhalb von 72 h), sie ist indiziert, wenn die Beatmung voraussichtlich länger als 12 Tage erforderlich ist.

Die perkutane endoskopische Gastrostomie (PEG) ist ein etabliertes endoskopisch chirurgisches Verfahren für Patienten mit Dysphagie. Sie sollte frühzeitig erfolgen, wenn auf absehbare Zeit oder auch dauerhaft nicht die notwendige tägliche Flüssigkeits- und Kalorienmenge oral aufgenommen werden kann oder schon vor der Antitumortherapie eine deutliche Untergewichtigkeit besteht. Indikationen für eine PEG-Anlage sind auch obstruktive oder neurogene Dysphagien mit reversiblen und irreversiblen Schluckstörungen sowie Aspiration [62]. Die Patienten können sich im Rahmen ihrer Möglichkeiten weiter oral ernähren. Der Zeitpunkt der PEG-Anlage ist häufig an die genannten Faktoren gekoppelt, einige Autoren empfehlen die prätherapeutische PEG-Anlage. Letztendlich gibt es bezüglich der kalorisch ausreichenden Menge Limitationen, weil die Sondenkost aspiriert werden kann, viele Patienten über Unverträglichkeiten und Meteorismus klagen und die erforderliche Menge dadurch nicht sondieren wollen. Hier kann zumindest ein Wechsel der Sondenkost auf z. B. ballaststoffreichere Ernährung Linderung verschaffen.

## Ausblick

Durch die demografische Entwicklung in Deutschland, die Assoziation von Schluckänderungen mit dem Alter und dem Auftreten von neurodegenerativen Erkrankungen werden sich Schluckstörungen allgemein häufen. Die heute individuellen Tumorbehandlungen im HNO-Bereich und das längere Überleben haben mehr tumorbehandlungsbedingte Schluckstörungen zur Folge. Die professionell-ärztliche Expertise darf sich bei dem Symptom Dysphagie nicht nur auf bemühte oder scheinbare kausale Ursachen stützen. Sie muss immer wieder identifizierte Ursachen kritisch neu hinterfragen und ggf. darüber hinaus Wissen außerhalb der eigenen Fachrichtung anwenden, um den Betroffenen ggf. korrigierte Empfehlungen zu geben und (veränderte oder neue) Entscheidungen treffen zu können.

## Fazit für die Praxis

- Schluckstörungen haben vielfältige Ursachen.
- Sie können in jedem Lebensalter bestehen oder beginnen, je nach funktionellem, anatomischem oder neurobiologischem Korrelat.
- Sie können zu erhöhter Mortalität bei Lungenkomplikationen und Mangelernährung einerseits sowie einer erheblichen Beeinträchtigung der Lebensqualität andererseits führen.
- Anamnese und Diagnostik erfordern eine interdisziplinäre Fokussierung auf das Symptom Dysphagie und einer nicht nur HNO-/phoniatrisch-spezifischen Expertisenbeurteilung, weil Schluckstörungen vieldimensionale Ursachen haben.
- Vorrangig ist die HNO-/phoniatrisch-ärztliche Expertise von Bedeutung in der Durchführung von onkologischen Operationen im Bereich der Schluckstraße unter möglichst großer Schonung der schluckrelevanten Strukturen sowie die Planung postkurativer, individueller Operationen zur Verbesserung des Bolustransports und der Vermeidung von Aspirationen.
- Die konservativen Möglichkeiten der Dysphagiebehandlung durch Ernährungsberatende, Logopäden, Physiotherapeuten und Ergotherapeuten komplettiert die interdisziplinäre kompetente und patientenzentrierte Betreuung.
- Für eine ganzheitliche Betrachtung kann am Lebensende auch die Entscheidung gegen eine PEG-Anlage/Ernährung und/oder eine Tracheotomie stehen.
